# Genetic Dissection of Dual Roles for the Transcription Factor *six7* in Photoreceptor Development and Patterning in Zebrafish

**DOI:** 10.1371/journal.pgen.1005968

**Published:** 2016-04-08

**Authors:** Mailin Sotolongo-Lopez, Karen Alvarez-Delfin, Carole J. Saade, Daniel L. Vera, James M. Fadool

**Affiliations:** 1 Department of Biological Science, The Florida State University, Tallahassee, Florida, United States of America; 2 Program in Neuroscience, The Florida State University, Tallahassee, Florida, United States of America; 3 Center for Genomics and Personalized Medicine, The Florida State University, Tallahassee, Florida, United States of America; New York University, UNITED STATES

## Abstract

The visual system of a particular species is highly adapted to convey detailed ecological and behavioral information essential for survival. The consequences of structural mutations of opsins upon spectral sensitivity and environmental adaptation have been studied in great detail, but lacking is knowledge of the potential influence of alterations in gene regulatory networks upon the diversity of cone subtypes and the variation in the ratio of rods and cones observed in numerous diurnal and nocturnal species. Exploiting photoreceptor patterning in cone-dominated zebrafish, we uncovered two independent mechanisms by which the *sine oculis homeobox homolog 7* (*six7*) regulates photoreceptor development. In a genetic screen, we isolated the *lots*-*of*-*rods*-*junior* (*ljr*^*p23ahub*^) mutation that resulted in an increased number and uniform distribution of rods in otherwise normal appearing larvae. Sequence analysis, genome editing using TALENs and knockdown strategies confirm *ljr*^*p23ahub*^ as a hypomorphic allele of *six7*, a teleost orthologue of *six3*, with known roles in forebrain patterning and expression of opsins. Based on the lack of predicted protein-coding changes and a deletion of a conserved element upstream of the transcription start site, a *cis*-regulatory mutation is proposed as the basis of the reduced expression of *six7* in *ljr*^*p23ahub*^. Comparison of the phenotypes of the hypomorphic and knock-out alleles provides evidence of two independent roles in photoreceptor development. EdU and PH3 labeling show that the increase in rod number is associated with extended mitosis of photoreceptor progenitors, and TUNEL suggests that the lack of green-sensitive cones is the result of cell death of the cone precursor. These data add *six7* to the small but growing list of essential genes for specification and patterning of photoreceptors in non-mammalian vertebrates, and highlight alterations in transcriptional regulation as a potential source of photoreceptor variation across species.

## Author Summary

Vision begins when an image is focused on the neural retina where rod and cone photoreceptors convert light into the electrical signals of the brain. The 4 cone subtypes in retinas of the majority of fishes, lizards and birds, provide rich color vision. In contrast, retinas of most mammals are better adapted for dim light conditions with rods vastly outnumbering the sparse and less diverse cone subtypes. However, our understanding of photoreceptor development largely based on findings from mammalian models fails to explain the tremendous diversity of cone subtypes and variation of rod and cone numbers across the majority of vertebrate species. Taking advantage of the cone-rich zebrafish retina, we identified a nuclear factor that suppresses the number of rods and is essential for the development of a cone subtype not present in mammals. Combined with prior studies, the findings provide insight into adaptive mechanisms underlying maintenance of a cone-dominated retina.

## Introduction

Sensory systems provide a critical link for an animal to its ever changing and complex environment. Retinal photoreceptors are the highly specialized neurons that transduce light into the chemical and electrical signals of the nervous system. Representatives from nearly all classes of extant vertebrates possess a duplex retina with two distinct types of photoreceptors: rods, which are highly sensitive to light, mediate scotopic or dim light vision; and cones, which function under daylight or bright light conditions, are responsible for color vision. The spectral sensitivity of cones is dependent upon the expression of one of four different visual pigments or opsins with peak sensitivity to ultraviolet or violet (SWS1), blue (SWS2), green (RH2), or red (LWS) wavelengths of light. Rods express rhodopsin (RH1) which is most sensitive to green light [1], [2]. Detailed phylogenetic and functional analyses of structural mutations affecting spectral sensitivity provide much insight about the evolution of the visual system and adaptation to different lighting environments [1], [2], [3], [4], [5], [6]. Nevertheless, the molecular mechanisms leading to the major evolutionary changes in photoreceptor composition among vertebrate species remain unclear.

Electrophysiological data provide compelling evidence that the first jawless vertebrates already possessed a duplex retina containing four cone subtypes as well as cells adapted to dim light conditions [7], [8],[9],[10],[11]. A cone rich architecture is still present in many extant species of teleosts, amphibians, reptiles, and birds [12], [13], [14], [15]; in stark contrast, retinas of nocturnal animals are typically rod-dominated and possess only one or two cone subtypes [16]. For example, the high number of rods, relatively few cones and eye shape reflect the prevailing view that Mesozoic ancestors of extant mammals were adapted to a nocturnal environment [17], [18], [19], [20], [21]. Today, the remaining cones in marsupials and eutherian mammals express LWS and SWS1 opsins, and in monotremes express functional LWS and SWS2 opsins [22], [23], [24]. The absence of RH2 and SWS2, but preservation of RH1, LWS and SWS1 opsins in the basal lineage of modern snakes is an example of convergent evolution to maintain short- and long-wavelength sensitivity in nocturnal or burrowing species; yet continued adaptation is observed in more recent gene losses, adaptation of additional sensory modalities or regain of trichromacy [24],[25],[26], [27] [28].

Retinal development proceeds in a highly conserved order with cones generated in the first wave of neurogenesis and rod generated later in development [29], [30]. The temporal difference is thought to represent a change in the competency of retinal progenitors over time [31], [32]. Phylogenetic analysis and experimental data support the notion that shifts in the timing of mitosis (heterochrony) are associated with alterations in the proportion of neuronal subtypes produced during retinogenesis [33]. For example, the greatly increased numbers of rod and bipolar cells in the nocturnal owl monkey (*Aotus azarae*) are associated with shifts in mitosis to later stages of development compared to a closely related diurnal capuchin monkey (*Cebus apella*) [34]. By comparison, analysis of mouse mutations and human diseases show that alterations in the photoreceptor gene-regulatory network leads to dramatic changes in the types and numbers of photoreceptors generated during development. The specification of photoreceptor precursors and subsequent expression of rod and cone specific genes requires the expression of the homeobox transcription factor *CRX* [35], [36], [37], [38]. Subsequently, *TRβ2* regulates the specification of the LWS cone [39], [40], and the transcription factor NRL and its downstream target NR2E3 act synergistically with CRX to specify the rod fate and drive rod gene expression [41], [42] while repressing expression of cone genes [43], [44], [45], [46], [47]. The roles of these transcription factors are highly conserved yet studies have failed to find evidence that alteration of this gene regulatory network drives adaptation of the visual system in different classes of vertebrates. In fact, little is known about the factors that generate the greater diversity of cone subtypes in non-mammalian vertebrates or the mechanisms underlying the wide range of rod to cone ratios in diurnal and nocturnal species [48], [49].

The spatial patterning of zebrafish photoreceptors combined with classical genetics and emerging gene-targeting technologies, offer unprecedented opportunities to investigate photoreceptor biology in a diurnal species [50], [51], [52], [53], [54]. Larval zebrafish retina contains four cone subtypes, which outnumber the far fewer, sparsely distributed rods. Previously, in a genetic screen, we identified a novel role for the transcription factor *tbx2b* in photoreceptor development. Mutations of *tbx2b*, a co-orthologue of *TBX2*, results in a cell fate switch of the SWS1 cone precursors into rods [53]. These data supported the conservation of the ontological relationship between the SWS1-expressing cones and rods in mammals and zebrafish, but challenged the notion of a default photoreceptor fate among species. Here, we report the characterization of a second mutation called *lots-of-rods junior* (*ljr*^*p23ahub*^) that results in an increased number and uniform distribution of rods in larvae but with little affect upon cones. Data provide strong evidence that *ljr*^*p23ahub*^ is a mutation in a *cis*-regulatory element of *six7*, a teleost member of the *sine oculis* family of homeobox transcription factors [55], [56]. Our previous data showed that knockdown of *six7* led to an increased number of rods, and Ogawa et al., (2015) reported increased rod gene expression and altered cone opsin expression in a *six7* knockout line [57], [58]. In this study, our genetic analysis using the hypomorphic allele and novel loss-of-function alleles reveal that *six7* independently regulates mitosis of photoreceptor progenitors in a dosage dependent manner and survival of green-sensitive cone precursors. In addition to expanding our knowledge of genes essential for maintenance of photoreceptor diversity in a diurnal species, the developmental variation in rod and cone numbers provide insight that may be informative for pursuing evolutionary steps from a cone-rich towards a rod-dominated retina.

## Results

### The *lots-of-rods-junior* locus regulates rod number and spatial patterning

**Fig 1:**
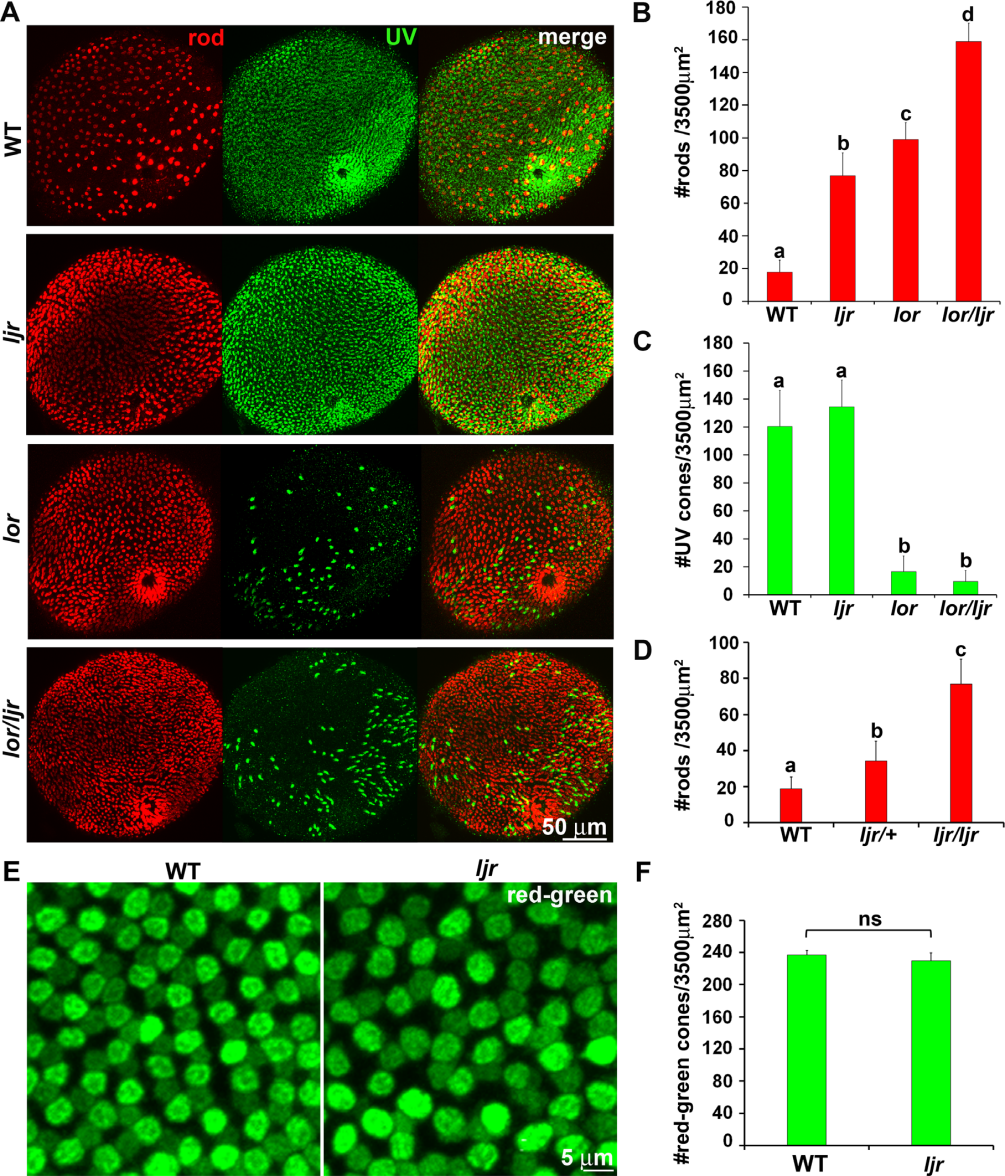
*ljr*^*p23ahub*^ mutants display an increased number and uniform distribution of rod photoreceptors. (A) Confocal immunofluorescent images labeled for rods (red) and UV-sensitive cones (green) from WT, ljr^*p23ahub*^, *lor*^*p25bbtl*^ and homozygous *lor*^*p25bbtl*^/ljr^*p23ahub*^ retinas at 4 days-post-fertilization (dpf). WT larvae show asymmetric rod distribution in central and dorsal retina and a uniform distribution of UV-sensitive cones across the entire retina. ljr^*p23ahub*^ and *lor*^*p25bbtl*^ mutants demonstrate an increased number and uniform arrangement of rods but fewer UV-sensitive cones labeled in *lor*^*p25bbtl*^. Double-mutant retinas display higher rod labeling and few UV-sensitive cones. (B) Graph showing the average number of rods per unit area dorsal to the optic nerve (WT, n = 5; *ljr*^*p23ahub*^, n = 5; *lor*^*p25bbtl*^, n = 5; *ljr*^*p23ahub*^*/lor*^*p25bbtl*^, n = 5). Significantly-different means by one-way ANOVA with Tukey’s post-hoc test, b vs c, p<0.05, all other comparisons p<0.0001. (C) Graph showing the average number of UV-sensitive cones per unit area from samples used in 1B, a vs b, p<0.0001. (D) Graph showing the average number of rods per unit area (WT, n = 5; *ljr*^*p23ahub/+*^, n = 6; *ljr*^*p23ahub*^, n = 5); a vs b, p<0.05, all other comparisons p<0.0001. (E) Flat mount views of confocal immunofluorescent images labeled for red-sensitive (brighter signal) and green -sensitive (dimmer signal) cones from WT and ljr^p23ahub^ retinas at 4 dpf. *ljr*^*p23ahub*^ mutants maintain the alternating arrangement of red- and green-sensitive cones. (F) Graph showing the average number of red- and green-sensitive cones per unit area (WT, n = 6; *ljr*^*p23ahub*^, n = 6). No significant differences are observed, Student’s *t* test, p>0.05. Error bars represent SD.

In our previously published genetic screen [53], [59] to isolate loci that regulate rod development and spatial patterning in zebrafish, we identified a mutation, *lots-of-rods-junior* (*ljr*^*p23ahub*^) that results in an increased number and uniform distribution of rods across the retina in otherwise normal appearing larvae. In wild-type (WT) larvae, rods are asymmetrically patterned along the dorsal-ventral axis of the eye, with the highest density in the ventral retina, few in a belt spanning the central retina, and sporadic, yet non-random labeling across the dorsal retina [60]. In homozygous *ljr*^*p23ahub*^ larvae, immunolabeling for rods results in a uniform distribution typical of the cone pattern (Fig 1A and 1B). For all mutant samples analyzed, conformity ratios (CR) were significantly different from random based on Cook’s criteria (p<0.05), and Nearest Neighbor Dispersion Analysis (NNDA) indicates the rods are arrange in a uniform pattern (p<0.05) [61]. Previously, we reported that mutations in *tbx2b* (*lor*^*p25bbtl*^) result in an increased number and uniform distribution of rods through a cell fate switch of SWS1-cone precursors into rods [53] (Fig 1A). Mating of homozygous ljr^p23ahub^ adults to homozygous *tbx2b*^*p25bbtl*^ adults revealed that *ljr*^*p23ahub*^ complements *tbx2b*^*p25bbtl*^; all larvae displayed a rod pattern and SWS1 cone number typical of WT larvae. Co-immunolabeled larvae from mating of the double heterozygous *ljr*^*p23ahub*^/*tbx2b*^*p25bbtl*^ adults revealed that double homozygous larvae for *ljr*^*p23ahub*^/*tbx2b*^*p25bbtl*^ displayed an additive number of rods and few UV-sensitive cones suggesting that these two genes regulate photoreceptor cell patterning through distinct mechanisms (Fig 1A, 1B and 1C). Furthermore, *ljr*^*p23ahub*^ is semi-dominant; heterozygous *ljr*^*p23ahub*^ larvae display a rod number between those of WT and homozygous *ljr*^*p23ahub*^ mutants (Fig 1D).

Whole mount *ljr*^*p23ahub*^ larvae and WT controls were immunolabeled for Zpr-1 monoclonal antibody, which recognizes Arr3a, a selective marker for the red- and green-sensitive cones [62]. In confocal images of flat mount WT retinas, Zpr-1-labeling appears as rows of cells with more intensely fluorescent red-sensitive cones alternating with less intense labeling of the green-sensitive cones (Fig 1E). No significant change in the number of Zpr-1 labeled cones in *ljr*^*p23ahub*^ mutant was observed when compared to WT (Fig 1F). Immunolabeling of serial sections of WT and *ljr*^*p23ahub*^ mutant larvae with polyclonal antisera to RH2, SWS2, and SWS1 revealed no differences in their expression levels (S1A Fig). No differences were observed for cell specific markers of amacrine, bipolar, ganglion or Muller cells in *ljr*^*p23ahub*^ compared to WT, suggesting a very specific retinal phenotype (S1B Fig).

### *ljr*^*p23ahub*^ as a hypomorphic allele of *six7*

**Fig 2:**
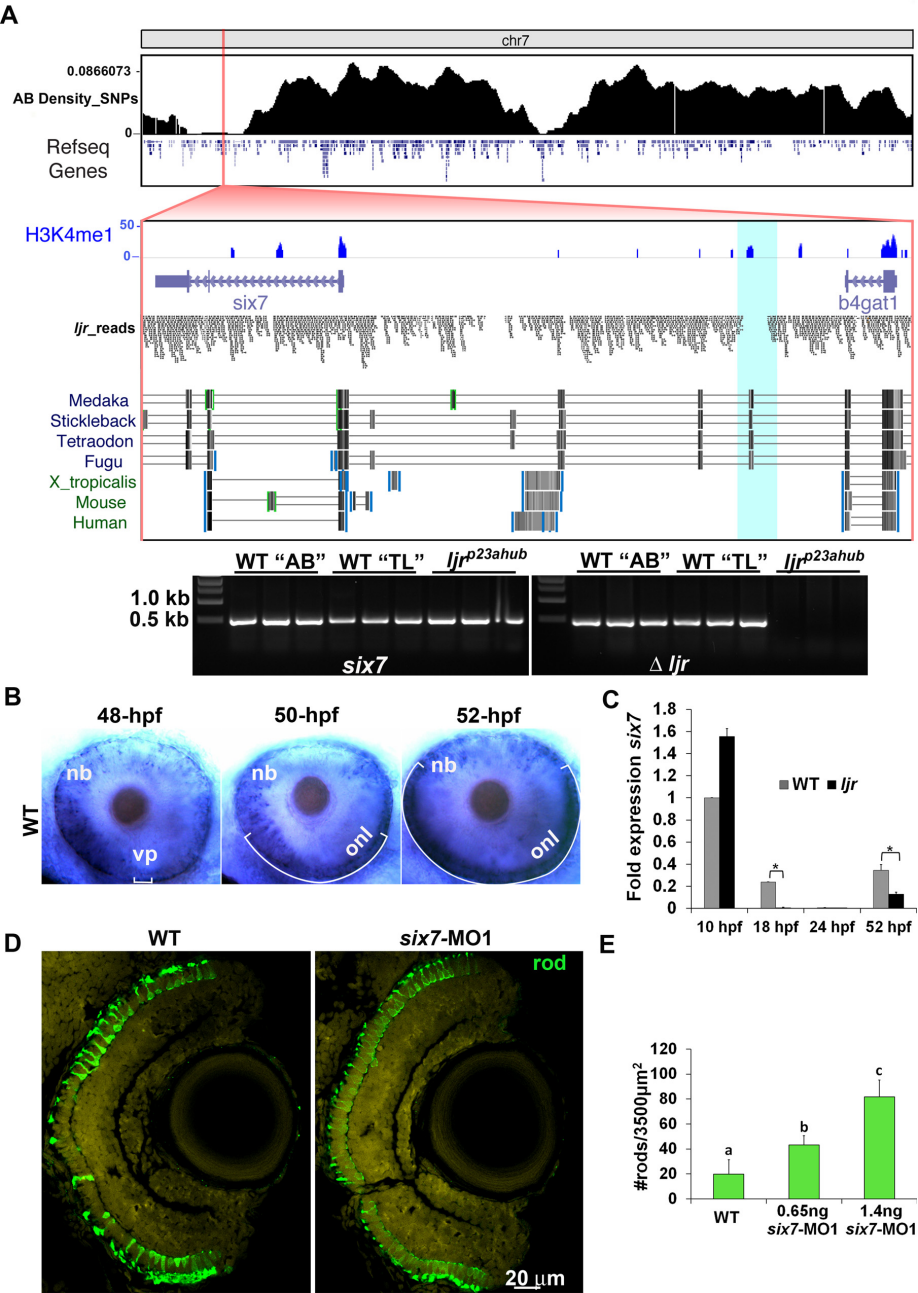
*six7* knockdown phenocopies the increase and uniform distribution of rods from *ljr*^*p23ahub*^ mutants. (A) Frequency of SNPs across chromosome 7 relative to the TL reference genome (danRer7) calculated from whole-genome sequencing of a pool of DNA extracted from 118 *ljr*^*p23ahub*^ mutants where TL is the reference background. Below, 75 kb view of the SNP-depleted region on chromosome 7. A 2.4 kb region depleted of uniquely-aligning reads is highlighted in teal. Shown are tracks for H3K4me1 [63], read alignments from whole-genome sequencing of *ljr*^*p23ahub*^ mutants, and multiz-based sequence conservation UCSC genome browser tracks of *six7* across four fish species and *Six3* in frog, human, and mouse [72]. Gel electrophoresis of PCR products of *six7* exon 1 and the upstream genomic region amplified from AB, TL and *ljr*^*p23ahub*^ genomic DNA. (B) Whole mount *in situ* hybridization shows *six7* expression confined to retinal neuroblasts and differentiating ONL spatially and temporally with photoreceptor genesis. Dorsal is up. (C) Quantitative RT-PCR (qRT-PCR) performed on mRNA from control (WT) and *ljr*^*p23ahub*^ embryos at 10 hpf, 18 hpf, 24 hpf and 52 hpf reveal down regulation of *six7* expression in *ljr*^*p23ahub*^ at 52 hpf. Relative transcript abundance was normalized to *actin* levels and is presented as the mean fold change in expression relative to 10 hpf controls (n = 30 embryos per group). All the real-time PCR experiments were carried out in triplicate. Significant differences observed at 18 hpf and 52 hpf, Student’s *t* test, *p<0.05. (D) Retinal cryosection from 4 dpf un-injected control WT and *six7*-MO1 injected embryos immunolabeled for rods (4C12, green). *six7*-morphants display an increase in the number of rods as detected in *ljr*^*p23ahub*^. Note the lack of gaps in rod distribution in the central retina of *six7*-knockdown larvae. (E) Graph showing dosage dependent increase in the average number of rods per unit area dorsal to the optic nerve of WT and *six7*-MO1 injected embryos (WT un-injected, n = 4; *six7-*MO1, n = 6, each dose), One-way ANOVA with Tukey’s post-hoc test. a vs b, p<0.05, b vs c, p<0.001, a vs c, p<0.0001. Error bars represent SD. nb, neuroblast; vp, ventral patch; onl, outer nuclear layer; MO1, morpholino 1.

Genetic linkage analysis positioned the *ljr*^*p23ahub*^ locus to a 0.3 Mb interval on chromosome 7 encompassing seven genes (S2A Fig): *Ras and Rab interactor 1* (*Rin1*), *actin related protein* (*Arp2*), *six7*, *beta-1*,*4 glucoronyltransferase 1*, *prefoldin subunit 2*, *nitrilase*, *and chloride channel 3*. Genes lacking eye specific expression (Zfin) or with housekeeping functions were excluded from further consideration leaving *six7* as the most plausible candidate. In order to more comprehensively examine for molecular lesions associated with the *ljr*^*p23ahub*^ we performed whole-genome sequencing of DNA pooled from 118 homozygous *ljr*^*p23ahub*^ larvae. Genome-wide SNP frequencies of reads were calculated against the Tuebingen (zv9) reference genome to identify regions depleted of SNPs that were associated with the mutant genetic background. Large, contiguous depletions in SNPs were present on chromosomes 3, 7, 9, and 20. A 5-megabase depletion of SNPs present on chr7 was centered directly over the mapping interval based on genetic linkage analysis (Fig 2A). Further examination of this interval revealed two regions, 14 kb and 40 kb upstream of *six7*, devoid of uniquely-aligning reads that were not associated with assembly gaps. The region 40 kb upstream of *six7*, but not the region 14 kb upstream, was also associated with sequence conservation among *six7* genes from 4 other fish species, but not with *Six3* from frog, mouse, or human. This region also displayed ChIP-seq signal for H3K4me1 in 24 hours-post-fertilization (hpf) embryos [63]. This mark is associated with distal enhancers in mammals [64]. To verify that the deletion was associated with the *ljr*^*p23ahub*^ mutation, DNA samples from fin clips of homozygous *ljr*^*p23ahub*^ adults and wildtype AB and TL strains of zebrafish were subjected to PCR using primer pairs targeting a DNA sequence unique to the distal deletion or the first exon of *six7* as a control. PCR confirmed the co-segregation of the genomic deletion upstream of *six7* with *ljr*^*p23ahub*^ (Fig 2A); the upstream region was only amplified in the TL and AB DNA. *six7* is a teleost specific member of the *sine oculis* homeobox family of transcription factors, which have important roles in eye and forebrain patterning [55], [65], [66], [67], [68]. In zebrafish, *six7* expression co-localized with *six3a/b* transcripts in the anterior region of the forebrain and optic vesicles [55], [69] with no detectable expression by RT-PCR at 24 hpf [55]. However, we and others observed by *in situ* hybridization that by 48 hpf, *six7* expression is detectable in the retina, specifically in neuroblasts and the differentiating outer nuclear layer (ONL) coincident with photoreceptor cell genesis [58] (Fig 2B). More precisely, between 48 hpf and 52 hpf more general labeling of the retinal neuroblasts gives way to robust *six7* expression that follows the temporal and spatial wave of photoreceptor genesis spreading from the ventral to the nasal and temporal retina [70], [71]. qRT-PCR for *six7* transcripts at developmental stages from 10 hpf to 52 hpf mirrored the *in situ* hybridization; in *ljr*^*p23ahub*^ mutant embryos, greater expression was observed at 10 hpf compared with WT, and expression was absent in *ljr*^*p23ahub*^ mutant embryos at 18 hpf and from both groups at 24 hpf. Lower levels in *ljr*^*p23ahub*^ mutant embryos at 52 hpf were observed (Fig 2C). Thus, the spatial and temporal pattern of *six7* expression and the changes observed in *ljr*^*p23ahub*^ embryos are consistent with previously described roles in photoreceptor development [57], [58].

To test the candidacy of *six7* as the mutated gene in *ljr*^*p23ahub*^, two antisense morpholinos targeting either the 5’UTR region (MO1) [69] or the donor splice site in the first intron (MO3) of *six7* were injected into one-cell-stage WT embryos (S2B Fig). Injection of either morpholino phenocopied *ljr*^*p23ahub*^ mutants (Fig 2D). Morphants showed increased rod immunolabeling in a dosage dependent manner (Fig 2E), and did not demonstrate any obvious morphological defects. To confirm the efficiency of the splice blocking morpholino, RNA was isolated from un-injected and MO3-injected embryos, and the region spanning exon 1 and exon 2 amplified by PCR from resulting cDNA. Sequence data revealed that the MO3-injections resulted in alternative splicing upstream of the initiation codon deleting the majority of the exon 1 of *six7* mRNA including the SIX domain (S2C Fig).

Previous studies reported highly conserved roles for *six3/six6* family members in patterning of the forebrain and eye field, and in zebrafish *six3b* and *six7* appear to be functionally redundant [58], [73], [74]. To study the hypothesis that *six3a/b* could be a target of *six7*, the levels of expression of *six3a/six3b* were determined by qRT-PCR. No difference in expression levels for *six3a/six3b* transcripts were detected between WT and *ljr*^*p23ahub*^ mutant (S2D Fig), suggesting that *six7* is not regulating the expression of *six3a/six3b*. Consistent with the expression of *six7* in *ljr*^*p23ahub*^ during forebrain patterning, analysis of 128 embryos from inbreeding of double heterozygous adults for *ljr*^*p23ahub*^ and *six3b*^*vu87*^ did not result in any embryos displaying a greatly reduced or absent eye phenotype as previously observed for *six7*-morpholino knockdown on the *six3b* mutant background or in the recently reported double mutant harboring deletions of *six7* and *six3b* [69],[58]. Based upon the genetic analysis, whole genome sequencing and gene expression changes, we propose that *ljr*^*p23ahub*^ is a hypomorphic allele of *six7* that affects a regulatory element controlling expression during photoreceptor genesis.

To further characterize the increased labeling for rods, cell counts from methylene blue stained plastic sections of 4-days-post-fertilization (dpf) *six7*-MO1 embryos revealed a modest yet significant increased number of cells in the ONL compared with WT retinas, consistent with the observed increased rod number (S2E Fig). However, no changes were detected in the number of nuclei in the inner nuclear layer (INL) or the ganglion cell layer (GCL), arguing against a general increase in neurogenesis across the retina (χ^2^, p> 0.05). Additionally, at 6 dpf, electron microscopy of the ONL in the central retina, where the highest change in rod numbers were detected, showed that rods in *six7*-MO1 were characterized by an outer segment composed of stacks of discs enclosed within the plasma membrane, a vitread located nucleus and a single invaginating synapse at the terminal (S2F Fig). These results suggest that knockdown of *six7* led to an increased number of retinal cells specifically in the ONL with gene expression and morphological characteristics consistent with rods.

### *six7* morphants show extended proliferation in the ONL

**Fig 3:**
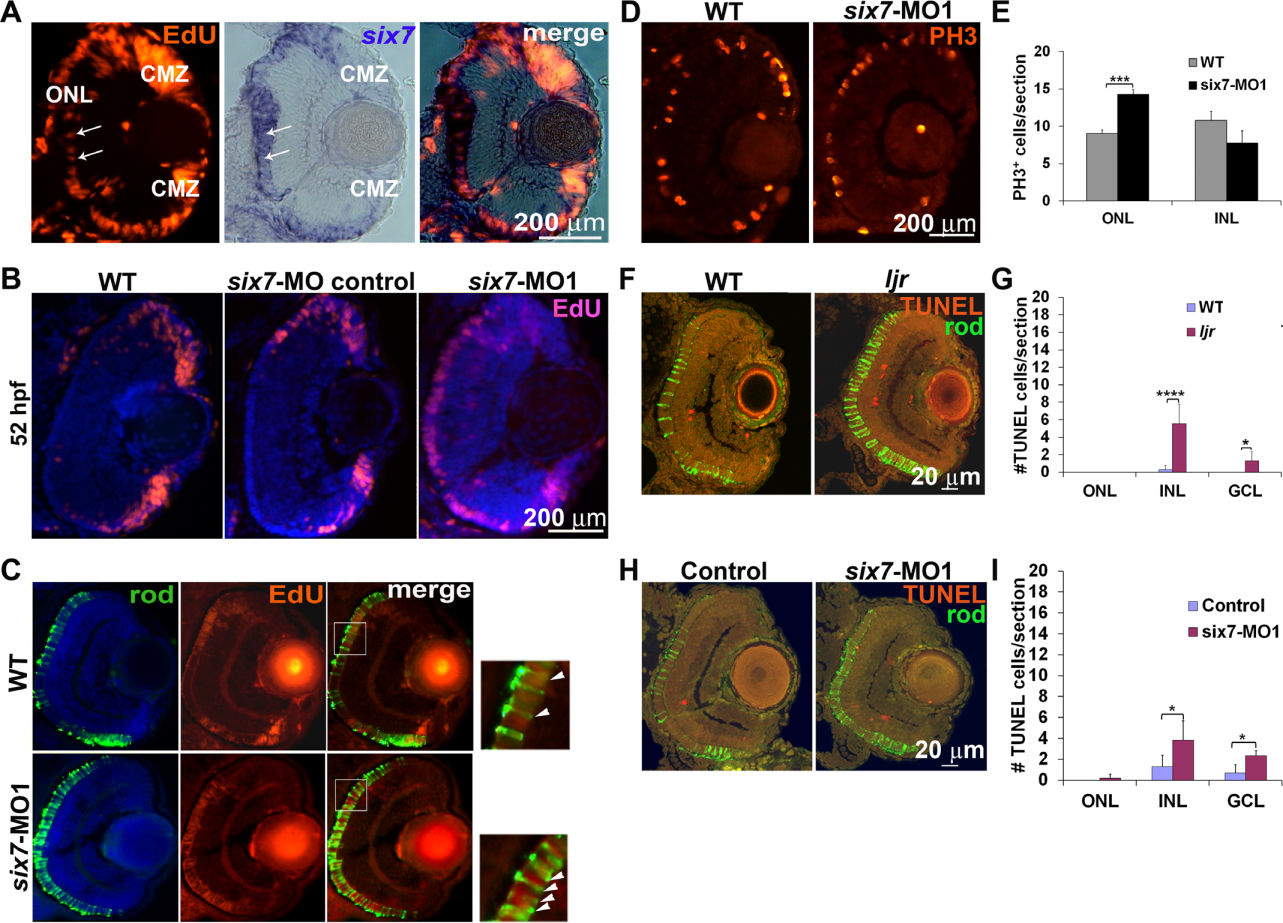
Photoreceptor progenitor proliferation is regulated by *six7*. (A) EdU (red) and *six7-in situ* labeling (blue) in a retinal cryosection from 48-hpf WT embryos. Note the expression of *six7* is coincident with proliferating cells in the ONL. (B) EdU labeling in retinal cryosections of WT, *six7*-MO control and *six7* morphants at 52 hpf. Note that EdU labeled cells persist in the central retina in *six7* morphants. (C) Retinal cryosections of WT and *six7* morphants labeled with EdU at 48 hpf and immunolabeled for rods (4C12, green) at 4 dpf. Proliferative cells are restricted to the ONL and differentiate as photoreceptors in WT and *six7*-knockdown retinas. Proliferative cells colabeled for rod markers in the central retina, (arrowheads, *inset*). (D) Retinal cryosections of WT and *six7*-morphants immunolabeled for phospho-histone 3 (PH3) at 48 hpf and (E) graph showing the number of PH3-positive cells in the INL and ONL per sections (WT, n = 5; and *six7*-MO1, n = 4; 3–4 sections/retina). Mitosis levels increased significantly in the ONL in *six7*-morphants. No significant changes were observed in the INL. (***p = 0.0007, Student’s *t* test). (F) Retinal cryosections of WT and *ljr*^*p23ahub*^ at 3 dpf co-labeled for TUNEL (red) and rods (4C12, green), nuclei counterstained with SYTO61. (G) Quantification of TUNEL positive cells in the ONL, INL and GCL of WT (n = 7) and *ljr*^*p23ahub*^ (n = 9); (*p<0.05, *****p<0.0001, Student’s *t* test calculated on log transformed data). (H) Transverse retinal sections from WT and *six7*-MO1 injected animals at 3-dpf co-labeled for TUNEL (red) and rods (4C12, green), nuclei counterstained with SYTO61. (I) Quantification of TUNEL positive cells in the ONL, INL and GCL of control (n = 7) and *six7*-MO1 injected embryos (n = 6); (*p<0.05, Student’s *t* test calculated on log transformed data). Error bars represent SD. Dorsal is up. ONL, outer nuclear layer; INL, inner nuclear layer; GCL, ganglion cell layer; CMZ, ciliary marginal zone.

The increased cell number in the ONL and lack of changes in cone numbers open the possibility that *six7* regulates mitosis during photoreceptor development. Proliferation was assayed by EdU incorporation or phospho-histone 3 (PH3) immunolabeling. In zebrafish, neurogenesis occurs in three distinct waves; postmitotic cells appear first in the GCL, followed by the INL, and finally the ONL. At 48 hpf, a time coincident with photoreceptor cell genesis [75], co-labeling for EdU incorporation and *in situ* hybridization for *six7* showed complex patterns of labeling. In histological sections, EdU labeling was most abundant in wedged-shaped clusters of highly proliferative cells near to the dorsal and ventral ciliary marginal zone (CMZ), and to a lesser degree in the developing ONL. In contrast, *six7* expression near the CMZ was opposite of the EdU labeling; highest proximal to the CMZ where the neuroblasts had taken on a more salt and pepper EdU-labeling pattern (arrows), and nearly absent from the highly proliferative CMZ. In the central retina, more robust labeling for *six7* coincided with reduced EdU incorporation (Fig 3A). These data show that *six7* is expressed in photoreceptor precursors at or near the time of terminal mitosis.

In a second set of experiments, un-injected, *six7* MO1-, and control morpholino-injected embryos were incubated with EdU at 48 or 52 hpf and immediately processed for labeling. At 48 hpf, all treatment groups showed EdU labeling in the ONL. However, at 52 hpf, *six7* MO1-injected embryos showed nuclear EdU labeling in the ONL but none was observed in the un-injected and 5 base mismatch control morpholino-injected embryos (Fig 3B). To determine if the EdU-labeled cells in the *six7*-morphant retinas differentiate specifically as rods, embryos were labeled with EdU at 48 hpf and maintained until 72 hpf, then fixed and processed for immunolabeling with a rod specific marker. EdU positive cells in the central retina co-labeled preferentially with a rod marker in *six7*-morphant retinas. However, some EdU positive cells differentiated as cones, consistent with the coincident timing of rod and cone differentiation in un-injected embryos (Fig 3C). We next immunolabeled retinas with anti PH3 to verify that the increased number of cells labeled with EdU was reflected by changes in the level of mitosis (Fig 3D). At 48 hpf retinas from *six7*-morphant embryos showed significant increases in the PH3 immunolabeling of the ONL compared to un-injected embryos (Fig 3E; Student *t*-test; p<0.001). No significant changes were observed in labeling of the INL. To test if the *six7*-depleted cells are biased to differentiate as rods, genetic chimeras were generated by transplanting cells from *six7* MO1-injected or WT donor embryos into WT hosts. In histological sections, *six7*-MO1 donors cells immunolabeled for a rod specific marker at a rate three times higher than WT transplanted cells (p<0.05; S3A Fig). The spatial and temporal appearance of the additional mitoses and bias to form rods led us to test the identity of the proliferative cells. WT and *six7*-knockdown retinas were labeled by *in situ* hybridizations with molecular markers for retinal progenitors, *rx1* and *pax6a* [76], [77] or two transcription factors expressed by developing photoreceptors, *crx* and *neurod* [78],[79],[80]. By 48 hpf in the WT and morphant larvae, the expression of the retinal progenitor marker *rx1* was restricted to the CMZ and the *pax6a* gene was expressed by neuroblasts of the CMZ and in neurons located in the GCL and the proximal portion of the INL [81]. None was observed in the ONL (S3B Fig). Probes for *crx* and *neurod* strongly label the forming ONL and to lesser extent cells of the INL, with no differences observed between WT and mutant larvae (S3B Fig). The data suggest that the mitotic cells are photoreceptor progenitors.

In lower vertebrates, such as zebrafish, cell death in the retina can trigger proliferation of Muller glia cells and photoreceptor regeneration [82], [83]. To exclude apoptosis-induced proliferation as the mechanism leading to the increase in rod number, sections from control, *ljr*^*p23ahub*^, and *six7*-morphant retinas were subjected to transferase-mediated dUTP nick end labeling (TUNEL) assay. Few TUNEL positive cells were observed in WT and control-morpholino retinas. A modest increased in TUNEL positive cells was observed in the inner retina and GCL in the *six7*-MO1 retina or *ljr*^*p23ahub*^ compared with control embryos (Fig 3F–3I), however none was observed in the ONL ruling out cell death-induced regeneration as the mechanism triggering the increase in mitosis and rod number. Lastly, previous studies in chick embryos have shown that ablation of the dorsal retina results in expansion of ventral domain and increased rod number in the central retina [84]. Given the high number of rods in the ventral patch of the zebrafish retina, we tested for expansion of ventral markers and loss of dorsal markers in *ljr*^*p23ahub*^ mutants. However, *in situ* hybridization for the dorsal marker *tbx2b*, the midline marker *cyp26c1*, and the ventral marker *vax2* showed no difference in labeling of WT and mutant embryos (S3C Fig), decreasing the likelihood that the increased rod number resulted from alteration of dorsal-ventral patterning of the optic cup.

### Genome editing of *six7* locus recapitulates the increased rod number in *ljr*^*p23ahub*^ mutant larvae

**Fig 4:**
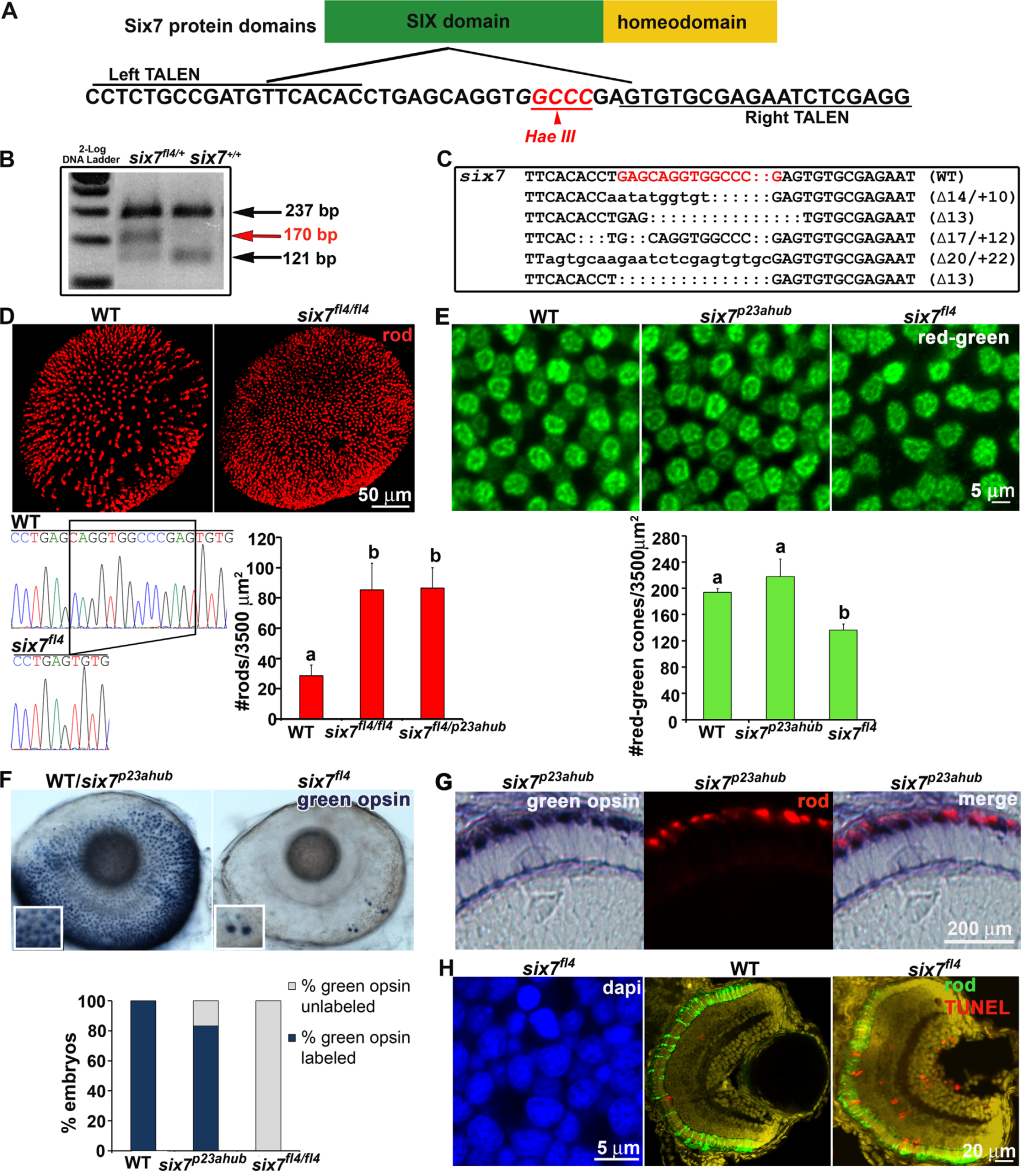
TALENs-mediated knockout of *six7* locus recapitulates *ljr*^*p23ahub*^ phenotype. (A) Schematic representation of the Six7 protein domains. *six7*-TALENs target site and the Hae*III* restriction enzyme site are highlighted. The left and right monomer binding sites are underlined. (B) RFLP of *six7* locus by Hae*III*. A new 170 bp DNA-fragment is detected in *six7*^*fl4*^ carriers compared with controls. (C) Sequence analysis of the *six7* target region shows recovery of multiple indel alleles of *six7*. The target region is highlighted in red. Dots represent deletions and lower case letters indicate insertions. The *six7* sequence from WT is shown as a comparison. (D) Confocal immunofluorescent images labeled for rods (red) from WT and *six7*^*fl4*^ knockout mutants at 4 dpf. *six7*^*fl4*^ knockout mutants phenocopy *ljr*^*p23ahub*^ mutants. Sequencing chromatograms of WT and *six7*^*fl4*^ mutants illustrated the c.217_229del CAGGTGGCCCGAG (del13) *six7* mutation in homozygous zebrafish. The bar graph shows the average number of rods counted in 1–3 different areas per retina (WT (n = 8), *six7*^*fl4*^ (n = 7), *six7* ^*fl4/p23ahub*^ (n = 4); One-way ANOVA with Tukey’s post-hoc test. a vs b, p<0.0001. (E) Tangential views of confocal immunofluorescent images labeled for red-sensitive (brighter signal) and green-sensitive (dimmer signal) cones from WT, *six7*^*p23ahub*^, and *six7*^*fl4*^ retinas at 4 dpf. Graph showing the average number of red/green-sensitive cones per unit area of WT (n = 3), *six7*^*p23ahub*^ (n = 4), and *six7*^*fl4*^ (n = 3); (ns p> 0.05; a vs b p ≤ 0.05; significant difference one-way ANOVA with Tukey’s post-hoc test). (F) Whole mount *in situ* hybridization for *RH2* probe (green-sensitive cone opsin) in WT (n = 30), *six7*^*p23ahub*^ (n = 30) and *six7*^*fl4*^ embryos (n = 30) at 4 dpf. WT and *six7*^*p23ahub*^ embryos often showed the same pattern and number of green-sensitive opsin cone labeling (Dorsal is up and nasal to the left), while *six7*^*fl4*^ knockout showed no labeling or few cells labeling for green-sensitive cone opsins (inset). Graph showing the percentage of unlabeled and labeled embryos. Notice that 17% of the *six7*^*p23ahub*^ embryos were un-labeled for green-sensitive cone opsin. (G) Retinal cryosections of *in situ* hybridization for green-sensitive cone opsin in *six7*^*p23ahub*^ (n = 5) embryos immunolabeled with 1D1 (rods). Rods and green-sensitive cone opsin probes labeled different cells in the ONL of *six7*^*p23ahub*^ embryos. (H) Evidence of cell death. Flat mount confocal image of nuclei counterstained with DAPI and retinal cryosections from WT and *six7*^*fl4*^ animals at 4dpf co-labeled for TUNEL (red) and rods (4C12, green). *six7*^*fl4*^ mutants (n = 6, 1–2 sections/retina) showed an increase in apoptotic cells, especially in the ONL compared with WT (n = 3, 1–2 sections/retina), (arrows pointing to apoptotic cells in the ONL).

Based upon the sequencing data, morpholino phenotypes and gene expression, we designed TALENs (transcription activator-like effector nucleases) to target *six7*. The homeodomain and the SIX domain are two evolutionarily conserved domains in the SIX proteins involved in DNA-protein or protein-protein interactions respectively [85], and mutations in the SIX domain in *SIX3* are associated with congenital brain and eye defects [86]. Therefore a TALENs pair was designed to target 18-bp and 20-bp flanking a 14-bp spacer sequence of the first exon of *six7*, which corresponds to the SIX domain (Fig 4A). mRNAs encoding for the TALENs pair were co-injected into one-cell stage zebrafish embryos. Surviving embryos were grown to adulthood and mated to WT adults. Disruption of the *six7* locus in the F1 larvae was detected by the loss of the *Hae*III restriction site in the spacer region (Fig 4B). Fifty-two percent of the founders transmitted TALEN-induced mutations to the F1 (Fig 4C). F1 progeny were grown to adults and heterozygous carriers identified by fin clip analysis. F2 carriers of the following alleles were used in subsequent studies: c. 217_229del CAGGTGGCCCGAG, p. (Q11Cfs*39), from now on (*six7*^*fl4*^); p. (E10Ifs*50); p. (F7Lfs*44), (all predicted to result in frameshift mutations and premature termination of the Six7 protein). Approximately, one quarter of the F2 progeny from inter-crosses between carriers demonstrated an increased number (*t*-test, p<0.001) and uniform distribution of rods (Cook’s CR, p<0.05; NNDA, p<0.05) as observed in *ljr*^*p23ahub*^ mutants (Fig 4D). Genotyping of the F2 larvae revealed that one quarter of the embryos with the *lots*-*of*-*rods* phenotype were heterozygous for the *six7*^*fl4*^ mutation, consistent with the semi-dominance previously observed in *ljr*^*p23ahub*^ mutants. In mating between carriers or homozygous mutant adults, *six7*^*fl4*^ failed to complement *ljr*^*p23ahub*^ mutants (Figs 4D and S4A; One-way ANOVA, Tukey’s follow-up test, p<0.0001); no significant difference in rod number was observed between *six7*^*fl4*^/*six7*^*fl4*^, *six7*^*fl4*^/*/ljr*^*p23ahub*^, and *ljr*^*p23ahub*^*/ljr*^*p23ahub*^ larvae (One-way ANOVA, p>0.5) providing genetic evidence that *ljr*^*23ahub*^ is indeed an allele of *six7*, and *ljr*^*p23ahub*^ shall be referred to as *six7*^*p23ahub*^. Lastly, as previously observed in *six7*-knockdown embryos, immunolabeling for PH3 showed significant difference in sections from *six7*^*fl4*^ homozygous embryos and WT siblings (S4B Fig). But unlike the *six7*^*p23ahub*^ allele, *six7*^*fl4*^ homozygous animals showed significantly reduced viability (*χ*^*2*^, p<0.05), but the few adults recovered were fertile.

### RH2 opsin expression and green-sensitive cone precursor survival are dependent upon *six7* expression

Ogawa et al. (2015) recently reported altered cone opsin expression in *six7* knock-out animals with RH2 expression nearly absent and SWS2 significantly reduced [58]. However, we observed few alterations with green-sensitive cone opsin expression in *six7*^*p23ahub*^ homozygous and *six7* morphant larvae and no changes in SWS2 immunolabeling. Therefore, we tested for differences in cone photoreceptor cell phenotypes between the *six7*^*p23ahub*^ and *six7*^*fl4*^ alleles. Immunolabeling and confocal analyses revealed a significant decline in the number of Zpr1/Arr3a positive photoreceptors in homozygous *six7*^*fl4*^ larvae consistent with the lack of RH2 expression reported previously (Fig 4E). Immunolabeled cells were evenly distributed across the retina, but clearly isolated from their neighbors as opposed to the mosaic of alternating red- and green-sensitive cones observed in the WT and *six7*^*p23ahub*^ mutant larvae (Fig 4E). *In situ* hybridization using an *RH2* probe or immunolabeling for green-sensitive opsin demonstrated that *six7*^*fl4/fl4*^ larvae were mostly devoid of green-sensitive opsin expression with only a few pairs of labeled cells in either eye (Fig 4F and *inset*); immunolabeling of histological sections confirmed the phenotype (S4 Fig). By comparison, homozygous *six7*^*p23ahub*^ mutants show variable penetrance of the loss of green-sensitive opsin expression phenotype; 83% of *six7*^*p23ahub*^ homozygous larvae showed a WT labeling pattern and only 17% showed labeling similar to the *six7*^*fl4*^ mutants (Fig 4F).Co-labeling with a rhodopsin antibody showed that all *six7*^*p23ahub*^ mutants retained the increased rod number regardless of the presence or absence of the green-sensitive opsin labeling (Fig 4G), and green-sensitive opsin positive photoreceptors do not co-label with several different rod markers, suggesting that the extra rods in the *six7*^*p23ahub*^ mutant retinas are not a rod-cone hybrid.

The similarities and differences in phenotypes of the hypomorphic and knock-out alleles from our lab and previously reported [58] suggest that *six7* regulates two distinct processes in photoreceptor cell genesis: terminal mitosis and differentiation or survival of green-sensitive cones precursors. Consistent with our hypothesis, confocal images of DAPI-labeled retinas from homozygous *six7*^*fl4*^ larvae showed gaps in the photoreceptor mosaic and small intensely labeled structures consistent with nuclei of dead or dying cells (Fig 4H). TUNEL was performed on *six7*^*fl4*^ retinas at 56 hpf, a time when expression of the all opsin subtypes should be detected [70], [87], and at 96 hpf, when all of the cones are mature. Few apoptotic nuclei were detected in WT or mutant retinas at 56 hpf. However, as development progressed to 96 hpf, considerable labeling of TUNEL positive cells was observed in the ONL of *six7*^*fl4*^ retinas (Fig 4H). Labeling was also observed in the INL and in fibers extending across both plexiform layers to the inner limiting membrane. The morphology and labeling pattern are consistent with that of Muller glial cells which have been shown to become TUNEL positive from phagocytosis of cellular debris following photoreceptor degeneration [88], [89], although we cannot rule out the possibility that a small population of cells in the inner retina is also dying. Together, the data show that *six7* is essential for development of green-sensitive cone precursors in the zebrafish retina and in its absence the precursors die.

### *six7* functions cell autonomously to regulate rod number and green-sensitive cone precursor survival

**Fig 5:**
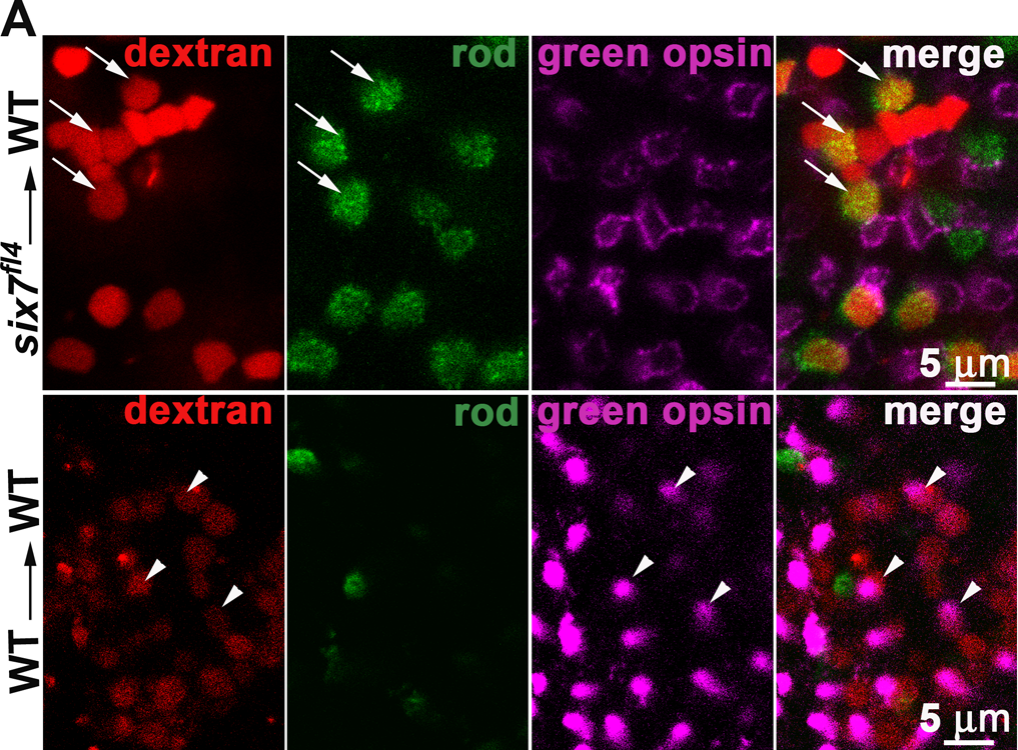
*six7* acts cell autonomously. (A) *six7*^*fl4*^-donor cells or (B) WT-donor cells were labeled by the tracer rhodamine-dextran, transplanted into WT genetic background and allowed to develop until 4 dpf. Expression of rods (4C12, green) and green-sensitive opsin (pink) were detected by whole mount immunolabeling. Note that *six7*^*fl4*^-transplanted cells frequently differentiate as rod photoreceptors (orange, arrows) and rarely immunolabeled for green-sensitive opsins while neighboring WT cells differentiate as green-sensitive cones. Significant difference was observed in the percent of *six7*^*fl4*^-donor cells that differentiate into rods or cones compared to WT donor cells (*χ*^*2*^, p<0.0001).

Genetic chimeras were generated to further test the cell autonomy of *six7* in photoreceptor biology. At blastula stage, cells were transplanted from rhodamine dextran-injected *six7*^*fl4*^ mutant donors into equivalent stage WT hosts. As control, WT cells were transplanted into WT embryos at the same developmental stage. The fate of WT vs *six7*^*fl4*^ donor cells showed statistically significant differences based upon co-immunolabeling host embryos for green-sensitive opsin and a rod marker (*χ*^*2*^, p<0.0001). Of 96 *six7*^*fl4*^ rhodamine positive donor cells in the ONL of 3 hosts, 49 cells (51%) co-labeled with a rod specific marker consistent with the data from the MO-injected genetic mosaics, but only 2 cells (2%) immunolabeled for green-sensitive opsin. However, the neighboring host cells frequently labeled for green-sensitive opsin (Fig 5A). In stark contrast, 16% of 106 rhodamine-labeled WT-donor cells co-labeled for the green-sensitive opsin, but only 5.6% for the rod-specific marker. These data are consistent with a cell-autonomous role of *six7* in regulating rod number and green-sensitive cone precursor differentiation or survival.

## Discussion

Taking advantage of photoreceptor patterning in the cone-rich, larval zebrafish retina, we characterize two independent roles for the transcription factor *six7* in photoreceptor development: *six7* regulates proliferation affecting the number and distribution of rods; *six7* is essential for survival of the green-sensitive cone precursor. We show that the increased number and uniform distribution of rods are associated with increased mitosis, and independent of and do not account for the loss of green -sensitive cones. Furthermore, the *six7*^*p23ahub*^ and *tbx2b*^*p25bbtl*^ double mutants show an additive number of rods indicative of activity in different pathways. Our research has identified genes essential for maintenance of a cone-dominated retina, and based upon the mutant phenotypes provide a context for understanding how alteration of *cis*-regulatory elements could drive developmental changes transitioning a cone-dominated retina to a rod-dominated retina.

The changes in rod number suggest that six7 has dosage-dependent affects upon mitosis. The heterozygous and homozygous mutant larvae displayed varying degrees of increased numbers of rods, and increased mitosis was observed in the ONL of mutant and *six7* knockdown embryos. In WT embryos *six7* is expressed in the ONL at 48 hpf, when few progenitors co-labeled for markers of proliferation, consistent with roles in the photoreceptor progenitors at or near the time of terminal mitosis. In zebrafish, gene expression studies, time lapse imaging and cell transplantation show that photoreceptor specification occurs prior to or coincident with cell cycle exit [58], [82], [90]. In *six7* mutants, the expression of *crx*, *neurod*, *pax6* and *rx1* were unchanged compared to WT animals suggesting that *six7* functions in mitotic photoreceptor progenitors downstream of *crx* and *neurod*. The proliferation phenotype in *six7* morphants and mutants was distinct from that observed in the *lep/ptc2* mutant larva which is characterized by a proportional increase in the number of neurons in each retinal layer [91]. Rather, the increase in rod number in *six7* mutants is consistent with the hypothesis that selective alterations in the timing of cell-cycle exit can vary the proportion of the retinal cell types produced [34],[92], [93]. The effects on mitosis are surprisingly different from those observed for *Six3*, the closest homologue for which data are available. In murine cortical progenitors, mis-expression of *Six3* caused clonal expansion, but the fate of cells could not be identified as the progenitors failed to differentiate [94]. Similarly, in the rat retina, retroviral-mediated ectopic expression of *Six3* led to an increased number of infected cells in the ONL relative to controls, though again the cells failed to mature properly. However, over-expression of a *Six3* variant that alters the protein binding domain resulted in nearly exclusive generation of differentiated rods. These data are consistent with a role for Six3/6/7 family members in cell cycle regulation but antagonistic to differentiation [95], The dissimilarities between *six7* and *Six3* may reflect inherent differences in the two protein, their binding partners, protein-protein interactions, or changes in the competency of the neural progenitors.

We initially reported that knockdown of *six7* resulted in an increased number of rods in the larval zebrafish retina, but no change in cone number was observed [57]. More recently, Ogawa et al. (2015) reported that TALENs-mediated knock-out of *six7*, in addition to increased expression of rod genes, resulted in loss of expression of *RH2* and lower expression of *SWS2*, however no mechanisms underlying these changes were identified [57] [58]. We show that these functions of *six7* in photoreceptor development are cell-autonomous. However, the differences in the phenotypes observed between the hypomorphic allele and loss-of-function alleles distinguish separate functions underlying the rod and cone phenotypes. In *six7*^*fl4*^ larvae, the virtual lack of labeling for *RH2*, gaps in the red- and green-sensitive cone mosaic and the presence of numerous TUNEL positive cells in the ONL are consistent with failure of the green-sensitive cone precursors to express markers of terminal differentiation and cell death. A few *six7*^*fl4*^ heterozygous larvae failed to label for *RH2*, and the phenotype was partially penetrant in larvae homozygous for the *six7*^*p23ahub*^ allele. This all-or-none labeling pattern suggests that a small but reproducible number of animals is sensitized to modest changes in the level of *six7* expression opening up the potential for genome sequencing data to reveal potential modifiers of the cone phenotype. In contrast, the observation of similar increased numbers and uniform spacing of rod in *six7*^*p23ahub*^ and *six7*^*fl4*^ larvae and the weaker phenotype observed in *six7*^*p23ahub*^ and *six7*^*fl4*^ heterozygous larvae suggests that rod number is quantitatively sensitive to changes in gene dosage. The ability to genetically separate the rod phenotype from the cone phenotype suggests that *six7* functions independently in the two populations of photoreceptor progenitors.

*six7* is the second gene we have identified which regulates rod and cone development in the zebrafish retina. We initially report a role for *tbx2b* in the specification of UV-sensitive cones. Although our results support the conservation of the ontological relationship between the UV-sensitive cones and rods observed in mammalian retinas, the identification of a novel role for *tbx2b* challenged the notion of a default photoreceptor phenotype. The subsequent identification of expression of *TBX2* in SWS1-expressing cones in chick suggests a conserved role in cone-dominated retinas [96]. The isolation of alleles of *tbx2b* and *six7* that show no change in coding sequence, but altered expression, provides insight into the potential for modulation of *cis*-regulatory elements as an underlying feature in varying the number and types of photoreceptors in some species [97]. Ogawa et al. (2015) speculated that genomic rearrangement led to *six7* acquiring a role in *RH2* expression in teleosts. Their phylogenic analysis predicts an early duplication event leading to the *six7*-subfamily but subsequent loss in birds and mammals. The evidence for a reptilian Six7 opens the possibility for a broader role for *SIX7* as many lizards also express a functional *RH2* [58]. *Cis*-regulatory alleles are considered unique players in phenotypic evolution [98], [99], [100], [101]. A basic tenant of the field of evolutionary developmental biology (Evo-Devo) is that small spatial or temporal changes in gene expression during development can have a dramatic effect upon morphology [102]. *Cis-*regulatory mutations are often co-dominant where natural selection operates more efficiently; heterozygous organisms express a new trait immediately rather than postponed until brought to homozygosity in the population [103], [104]. Frequently, mutations in *cis*-regulatory sequences are modular in their effect, leading to alleles with reduced pleiotropy, which would be favored over structural changes in individual proteins which would risk loss of essential functions in the intermediate phenotypes. Lastly, selection would necessitate that the output of the system is sensitive to variations in the level of expression of the factors. The alleles we recovered show many if not all of these features.

We propose that changes in the photoreceptor gene-regulatory network are one potential source for adaptive changes in rod and cone numbers in evolution. This and our previous study of *tbx2b* identified distinct mechanisms for maintaining the cone-dominated retina in a diurnal species. The mutant phenotypes are consistent with the previously proposed evolutionary trajectories that may have been associated with the adaptation to a nocturnal environment although the precise mechanisms remain to be discovered. Based upon phylogenetic analysis and environmental considerations, Davies et al., (2012) proposed a series of structural mutations in opsins associated with adaptation to the present day, rod-dominated phenotype of extant mammals [23]. Similarly, the loss of RH2 and SWS2 are observed in the basal lineage of snakes [25]. However, diurnal or nocturnal vision is not merely defined by the expression of a specific opsin, but rather by the coordinated expression of signal transduction genes, metabolic function and structural elements to maximize sensitivity or spatial and temporal resolution. Additively, increased mitosis of late stage progenitors, selective loss of specific opsin subtypes, and mutations of *cis*-regulatory enhancers could dramatically alter the types and ratios of photoreceptors in the retina. It is worth mentioning that evidence suggests modification of a rod into a middle wavelength-sensitive cone-like photoreceptor in the recent evolution of the all-cone retina of garter snake [105]. Thus, a few genetic changes could result in a significant shift of photoreceptor composition to retinas better adapted for a novel environment.

Characterizing the *cis*-regulatory elements and *trans*-acting factors are essential steps towards a more complete understanding of the mechanisms regulating the variations in photoreceptor numbers in zebrafish, and how the potential conservation or loss of these mechanisms shapes photoreceptor patterning in other species. Regardless of the exact mechanism, our study clearly indicates the potential of a small number of genotypic changes in a gene regulatory network provide substantive developmental alterations in photoreceptor genesis.

## Materials and Methods

### Zebrafish lines and maintenance

Zebrafish (*Danio rerio*) were reared, bred and staged according to standard methods [106]. *ljr*^*p23ahub*^ was isolated from a three-generation screening of N-ethyl-N-nitrosurea-mutagenized zebrafish immunolabeled for rods at 5 dpf [88]. Mutagenesis was performed at the University of Pennsylvania as previously described [107]. The *lor*^*p25bbtl*^ mutant was previously characterized [53]. The *six3b*^*vu87*^ mutant was previously described [69] and was generously provided by Dr. Solnica-Krezel (Washington University, St. Louis, MO). All animal procedures were approved by the Florida State University (FSU) Institutional Animal Care and Use Committee, ACUC Protocol #1421. Animals were anesthetized using MS222 and euthanized in ice water.

### Mapping and sequencing

*ljr*^*p23ahub*^ mutant embryos from mating heterozygous adults were identified by immunolabeling as previously described [53]. Linkage mapping was performed at the Zebrafish Mapping Facility at the University of Louisville from DNA isolated from 100 *ljr*^*p23ahub*^ mutant- and 100 WT sibling embryos using simple sequence-length polymorphism markers. Fine resolution mapping was performed with 463 *ljr*^*p23ahub*^ mutant embryos [108]. Genomic DNA from 118 *ljr*^*p23ahub*^ mutant embryos was isolated (DNeasy Blood 7 Tissue Kit; Quiagen, Valencia, CA, USA) and used for Illumina sequencing at the University of Texas Genomic Sequencing and Analysis Facility as previously described [109]. Reads were aligned to the zv9 Zebrafish genome assembly (ensembl) with BWA [110] using default parameters. Reads with alignment quality of at least 30 were used identify SNPs against the zv9 genome assembly using samtools mpileup and bcftools 0.1.19 [111]. SNP densities were calculated using bedtools2 [112]. Data was visualized using the UCSC genome Browser [113], [72]. Genomic DNA was isolated from tail-clip of adult zebrafish and the candidate deleted region was confirmed by PCR in: *ljr*^*p23ahub*^ mutants (n = 9) and WT embryos from: AB genetic background (n = 6), TL genetic background (n = 6) using the primers listed in S1 Table. PCR of *six7* fragment was used as positive control using primers listed in S1 Table.

### Morpholino injections

One of three different morpholinos (MO) were injected into one-cell stage WT embryos (Gene Tools, LLC, Philomath, OR): mispaired-control MO, 5’-CGAACGCCATTCCGAGTCTGACTAAC-3’; antisense nucleotide targeting *six7* 5’-UTR (MO1),5’-CCAACGGCATTCCAGTGTGAGTAAC-3’ [73]; and *six7* splice-blocking MO (MO3), 5’-GTACTTTTTGGTCTCACCTTAAAGC-3’. Unless otherwise stated, embryos were injected with 0.87 ng of the indicated MO. To confirm the efficiency of MO3, RNA was isolated from un-injected and MO3-injected embryos and the region spanning from exon 1 to exon 2 of the *six7*-transcript was amplified by PCR using primers listed in S1 Table. The truncated *six7*-transcript was sequenced using Applied Biosystems 3730 Genetic Analyzer with Capillary Electrophoresis (Foster City, CA).

### Genome editing by TALENs

TALEN expression vectors were constructed in the Mutation Generation and Detection Core, University of Utah to target the exon 1 of *six7* transcript. DNA plasmids were linearized by NotI (Invitrogen, Carlsbad, CA) and used as templates for TALEN mRNA synthesis with SP6 mMESSAGE mMACHINE Kit (Ambion, Austin, TX). To target the *six7* genomic sequence, 50–200 pg of the pair of TALEN mRNAs were injected in one-cell stage zebrafish embryos. Injected embryos were raised to adulthood and crossed to WT animals to generate the F1. DNA was extracted from either F1 embryos (groups of three to six embryos) from the outcross of founders or tail clips from adult F1 fish. To screen for insertions and deletions (indels), DNA was extracted and used as the PCR template to amplify the *six7*-TALENs targeted region using primers listed in S1 Table. The DNA fragment was subjected to restriction fragment length polymorphism (RFLP) assay. Indels were tracked by loss of *Hae*III (Invitrogen) restriction enzyme site in the targeted region. PCR products were sequenced to characterize the indels. The F1 embryos of positive founders were intercrossed to generate the F2 generation. F1 and F2 embryos were fixed in 4% paraformaldehyde in 80% phosphate-buffered saline (PFA/PBS) and processed for whole-mount rod immunolabeling as described (see Immunohisto-and immunocytochemistry).

### Real-time quantitative PCR

RNA extraction was performed in TRIzol (Invitrogen) from pool of whole embryos (n = 30) at 10 hpf, 18 hpf, 24 hpf and 52 hpf. Transcription into cDNA was performed using SuperScript™ II Reverse Transcriptase (Invitrogen). Real time quantitative PCR (RT-qPCR) was carried out using a 7500 Real-Time PCR Systems (Applied Biosystems) with SRBY-Green PCR Master Mix (Applied Biosystems) and the primers listed in S1 Table. Three biological replicates were performed for each developmental time and were duplicated for each cDNA sample for *six7* qRT-PCR. The fold expression change was normalized to *β-actin* using the 2^-∆∆CT^ (Livak) method [114]. Student’s *t* test was applied for comparison between groups at each developmental time.

### Immunohisto-and immunocytochemistry

Immunolabeling of larvae whole mount or cryosections (10 μm) was performed as previously described [88]. Sections and enucleated eyes from whole-mounted immunolabeled larvae were imaged using either a Zeiss Axiovert S100 fluorescent microscope (Carl Zeiss Inc., Thornwood, NY) or a LSM 510 or LSM 710 (Carl Zeiss) Laser Confocal equipped with a 40x C-Apochromat water immersion objective (N.A. 1.2). The following primary antibodies were used: monoclonal antibody 4C12 that labels rods (1:200, [115]), a monoclonal antibody zpr1 that labels double cone cells (*arr3a*) (1:20, [62]), a monoclonal antibody 1D1 against rhodopsin [115] a polyclonal antibody against zebrafish blue-, red-, green- or UV-sensitive cone opsin (1:200, [12]), Zn8 which recognizes ganglion cells (1/10, ZIRC), 5E11 that labels amacrine cells ([115], PKCα that labels bipolar cells (1/100, [57]), CAZ which recognizes Muller glia (1/100, [116]) and polyclonal PH3 antibody that labels mitosis marker phospho-Histone 3 (1:500; Cat. No. 06–570, Millipore, Billerica, MA). Host-specific, Alexa fluor-conjugated secondary antibodies (Invitrogen) were used at a dilution of 1:200. Sections were counterstained with 4′,6-diamidino-2-phenylindole, dihydrochloride **(**DAPI, 1:15000; Sigma-Aldrich).

Proliferation was assessed by incubation of 48 hpf and 52 hpf embryos in fish water with 1.5 mM EdU (5-ethynil-2’-dexyuridine) during 30 minutes and subsequently fixed in 4% paraformaldehyde (PFA/PBS). The EdU labeling was processed by the Click-iT EdU Alexa Fluor 546 Imaging kit (Invitrogen) following the manufacturer’s instructions. For lineage tracing cell experiments, the EdU was washed with fish water and the embryos were incubated until 4 dpf and subjected to immunohistochemistry.

### Light and transmission electron microscopy

Larvae were euthanized with tricaine and processed according to the protocol by [117], with slight modifications. Briefly, larvae were fixed overnight with 1% glutaraldehyde and 1% osmium tetroxide in 0.1 M cacodylate buffer. The eyes were then washed 3 times and dehydrated through a graded (70%, 75%, 90%, 100%, 100%, 100%) acetone-water series. The tissue was infiltrated overnight at room temperature in a 1:1 mixture of epoxy resin and 100% acetone. Larvae were then embedded in epoxy resin and placed in a 60°C oven for 22 hours. 1μm sections were cut with a microtome and mounted on glass slides and stained with 1% methylene blue in 1% sodium borate. Photomicrographs were taken with a Zeiss Axiovert microscope, and images were captured by the Zeiss Axiocam Digital Camera and processed using the Axiovision software. For electron microscopy, sections were collected on copper grids and viewed with a FEM CM 120 transmission electron microscope. Electron micrographs were taken using a Tietz Tem-Cam F224 slow scan CCD camera, prior to being imported into Photoshop version 8.0 (Adobe Systems).

### Quantitative analysis

Confocal images from whole eyes immunolabeled for UV-sensitive cones and rods were analyzed with the Scion Image Software (Scion Corp, Frederick, MA). Areas of 3500 μm^2^ located dorsal to the optic nerve [53] were counted for rods and UV-sensitive opsin expressing cones in WT (n = 5); *lor*^*p25bbtl*^ (n = 5); *ljr*^*p23ahub*^ (n = 5); double mutant *lor*^p25bbtl/ljrp23ahub^ (n = 5) at 4 dpf. Quantification of rods was conducted similarly as for WT (n = 5); *ljr*^*p23ahub*^ (n = 5) and *ljr*^*p23ahub/+*^ (n = 6) an independent experiment. In addition, rods were similarly counted in WT (n = 8); *six7* ^*fl4/fl4*^ (n = 7), *six7* ^*fl4/p23ahub*^ (n = 4). The number of red-green-sensitive cones was quantified in WT (n = 6); *ljr*^*p23ahub*^ (n = 6); and in independent experiment WT (n = 3); *six7* ^*p23ahub*^ (n = 6); *six7* ^*fl4*^ (n = 3). When possible two of 3500 μm^2^ retinal areas were counted. *six7*-MO1 injected retinas (n = 5–6 retinas/each MO1 dose) were imaged and rods were quantified at 4 dpf for 3500 μm^2^ area. Un-injected (n = 4) retinas were used as controls. The average number of UV-, Arr3a-positive or rod-positive cells per unit area and the standard deviation (SD) were reported. One-way ANOVA with Tukey’s post-hoc test was used to compare means of rods or UV-sensitive cones between different genotypes. Student *t* test was applied to compare two sample data.

The number of PH3 positive cells in the ONL and INL was quantified using 10-μm-thick-retina sections of 21 000 μm^2^ area per section that excluded the CMZ. The following strains and number (n) of 48-hpf retinas were analyzed: WT (n = 5), *six7*-MO1 injected embryos (n = 4). A Student’s *t*-test was conducted to compare the number of PH3 positive cells in ONL and INL between WT and *six7*-knockdown retinas. Same procedure was used to count PH3 positive cells in the ONL and INL of *six7* ^*fl4/f+*^ (n = 5) and *six7* ^*fl4/fl4*^ (n = 11). Un-paired Student *t* test with Welch’s correction was used for statistical analysis.

Quantitative analysis of photoreceptor pattern was performed as described [59]. Nearest Neighbor Dispersion Analysis (NNDA) was determined using Biotas and the conformity ratio was calculated and analyzed for randomness using the Ready-Reckoner Chart of Cook [61]. Fluorescent structures were assigned (*x*, *y*) coordinate using ImageJ software (National Institutes of Health Windows version, (http://rsbweb.nih.gov/ij/index.html). For each point in the field Nearest Neighbor Distance (NND) was calculated using Biotas (Version 1.02; Ecological software Solutions).as previously described [59].

### Whole-mount *in situ* hybridizations

Whole-mount *in situ* hybridizations were performed as previously described [118] using pools of 25 embryos at 28 hpf and between 46–52 hpf. The antisense riboprobes were: *six7* (this study), *vax2* (this study), *cyp26c1* (this study) and *tbx2b* [53]. The plasmids containing the probes for *neurod* [80], *crx* [76], *pax6a* [119], *rx1* [120] were kindly provided by A.C. Morris (University of Kentucky, Lexington, KY). To prepare a probe for *six7*, a 444 bp fragment of *six7* gene was amplified from a cDNA fragment obtained from 10 hpf embryos, using primers listed in S1 Table and cloned into the vector PCR2.1-TOPO (Invitrogen). Antisense RNA probe was synthesized with a digoxigenin RNA-labeling kit (Roche, Indianapolis, IN) by *in vitro* transcription with T7 RNA polymerase, according to the manufacturer's instructions. A 620 bp of *vax2*-cDNA fragment and a 692 bp of *cyp26c1 were* amplified using primers listed in S1 Table. The antisense probes were prepared as described above. The hybridized probe was detected with alkaline phosphatase coupled with anti-digoxigenin antibodies and NBT/X-phosphate substrate (Roche). Labeled embryos were cleared in a graded series of glycerol and viewed on a Zeiss Axiovert S100 microscope. Images were captured by Carl Zeiss Axiocam Color Microscope camera and processed with Axiovision SE64 Rel 4.9.1 and Photoshop 5.5 (Adobe, Mountain View, CA) software.

### TUNEL assay

Terminal deoxynucleotide transferase (TdT)-mediated dUTP nick labeling (TUNEL) was performed on 3 dpf retinal cryosections using the ApopTag Red *In Situ* Apoptosis Detection Kit (Millipore, Temecula, CA) according the manufacturer’s instructions and co-labeled for rods (4C12) to identify mutants. TUNEL positive cells were counted in the ONL, INL and GCL from: WT (n = 7); *ljr* (n = 9); control (n = 7) and *six7*-MO1 (n = 6), one section for individual embryo. TUNEL positive cell counts were transformed (log Y+1) before student *t* test was conducted. TUNEL assay was performed in *six7*^*fl4*^ mutants and WT embryos at 56 hpf and 4 dpf. Tail-clip genotyping was used to identify mutants at 56 hpf. The following strains and number (n) of 56 hpf were analyzed: WT (n = 5), *six7*^*fl4*^ (n = 7) and at 4 dpf: WT (n = 3), *six7*^*fl4*^ (n = 6).

### Cell transplantation

Genetic chimeras were generated as previously described [121]. Donor embryos were injected at the 1- and 2-cell stage with the lysine-fixable, dextran-conjugated Alexa Fluor 594 (Invitrogen). Donor blastulae cells were transferred to unlabeled host cells. At 4 dpf the chimeras were fixed with 4% PFA/PBS and immunolabeled for rods and green-opsin as described above. Imaging of the whole-dissected eyes was performed by confocal microscopy (WT into WT, n = 3; *six7*^*fl4*^ into WT n = 3; WT into WT, n = 5; *six7*-MO1 into WT, n = 6). The number of rhodamine-dextran labeled cells, 4C12/dextran (rod from donor cells)-labeled and green-opsin/dextran labeled cells were quantified. The percentage number of donor cells differentiated as rod photoreceptor or green-sensitive cones were compared for *six7*^*fl4*^ mutants vs WT transplants into WT background. Numbers of donor cells were counted across the retinal layers from retinal sections of *six7*-MO into WT. Statistical analysis was performed by chi-square test.

## Supporting Information

S1 Fig **(A) No changes in green-, UV- and blue-sensitive cone opsin expression in *ljr***^***p23ahub***^ **mutants**. Retinal cryosections from WT and *ljr*^*p23ahub*^ embryos at 4 dpf immunolabeled for rods (4C12, red) and the green-, UV- and blue-sensitive cone opsins (green). Nuclei were counterstained with DAPI (blue); dorsal is up. No differences in the number or expression levels are detected for any of the opsin subtypes, except for the increased number of rods in *ljr*^*p23ahub*^ mutants. (B) retinal cryosections from WT and *ljr*^*p23ahub*^ embryos at 4 dpf immunolabeled for ganglion cells (Zn8), amacrine cell (5E11), bipolar cells (PKCα) and rods (4C12). No differences were observed for any labeled retinal cell types.
(TIF)

S2 Fig *six3a/b* expression is unchanged in *six7* morphants. (A) Linkage analysis places the *ljr*^*p23ahub*^ locus on chromosome 7, 6 out of 463 larvae show recombination at marker ZC168D1-SSR6 and 1 out of 252 larvae show recombination at marker ZC42C5-SSR2. (B) Diagram of predicted morpholino recognition sites (bars) in *six7* loci. MO1 targets a translational site and MO3 blocks a donor splice site in intron 1 of *six7*. Incorrect splicing can be seen in morpholino-injected animals using primers in exon1 and 2 (arrows). (C) RT-PCR fragments, using primers highlighted in A, were analyzed by 1% agarose gel electrophoresis. Arrow highlights the *six7* alternative spliced product obtained at 12 hpf (* indicates the new cryptic splice site). (D) No changes in the expression of the homologues *six3a* (left graph) or *six3b* (right graph) were detected between WT and *ljr*^*p23ahub*^ mutants (n = 30 embryos per group). All the real-time PCR experiments were carried out in triplicates and normalized to β-actin. (E) Plastic sections of 4 dpf retinas from WT and *six7*-MO1 injected larvae. The three layers are regularly arranged in WT and morphant retinas. Close examination of the retina revealed a densely packed ONL in morphants. Graph showing the average number of nuclei per unit area (WT, n = 3, 2 sections each; *six7*-MO1, n = 3, 2 sections each). Cells are increased in the ONL of *six7* morphants. Student *t* test, arcsine transformation, *p<0.05. (F) Electron micrographs of rod outer segments (ROS) from central retina of WT and *six7*-MO1 larvae. ROS from six7 morphants show their typical morphology of parallel- flattened sacs with continuously membranes at the edges (arrows) indistinguishable from WT ROS.
(TIF)

S3 Fig *six7* functions in photoreceptor progenitor cells. (A) Histological sections of chimera retinas labeled for rods (4C12, green). *six7*-MO donor cells (red) preferentially generate rods compare to WT donor cells. Note the gap in rod labeling in WT/WT controls. Graph represents the percentage of donor cells that differentiate as rods in the central retina of WT/WT (n = 5) and *six7*-MO1/WTchimeras (n = 6). *p<0.05, student *t* test. (B) *rx1*-, *pax6a*-, *crx*- and *neurod-in situ* hybridization (blue) in a retinal cryosection from 48-hpf WT and *six7-*MO1 embryos. No labeling of retinal progenitors cell markers (rx1 and pax6a) were observed in the ONL of *six7*-morphant retinas. (C) Whole-mount *in situ* hybridization for the dorsal (*tbx2b*), midline (*cyp26c1*) and ventral (*vax2*) retinal marker. Labeling was indistinguishable different between WT and mutant embryos.
(TIF)

S4 Fig Levels of mitosis are altered in *six7*^*fl4*^ at 56 hpf. (A) Confocal immunofluorescent images labeled for rods (4C12, red) from WT, *six7*^*fl4*^ and *six7*^*fl4/p23ahub*^ retinas at 4 dpf. (B) Retinal cryosections from carrier animals (*six7*^*fl4*^/+, n = 5, 1–2 sections/retina) and *six7* (n = 11) embryos at 56 hpf co-labeled for TUNEL (red) and PH3 (green), nuclei counterstained with DAPI. No differences in TUNEL labeling were detected. Graphs showing the number of PH3^+^ cells by section (excluding CMZ). Number of PH3^+^ cells is significantly greater in ONL of *six7*^*fl4*^ mutants at 56 hpf. Un-paired Student *t* test with Welch’s correction, *p<0.05). (C) Retinal cryosections from WT and *six7*^*fl4*^ embryos at 4 dpf immunolabeled for rods (4C12, red) and the green-, UV-, blue- and red-sensitive opsins (green). Nuclei were counterstained with DAPI (blue). Dorsal is up. Depleted green-sensitive opsin expression is noticeable in *six7*^*fl4*^, but other opsins appear unaltered.
(TIF)

S1 Table Primer sequences. (RTF)
