# Supplementary figures and images for: Genetic Dissection of Dual Roles for the Transcription Factor six7 in Photoreceptor Development and Patterning in Zebrafish
Source: PLoS Genet. 2016 Apr 8;12(4):e1005968. doi: 10.1371/journal.pgen.1005968 (PMC4825938; doi:10.1371/journal.pgen.1005968)

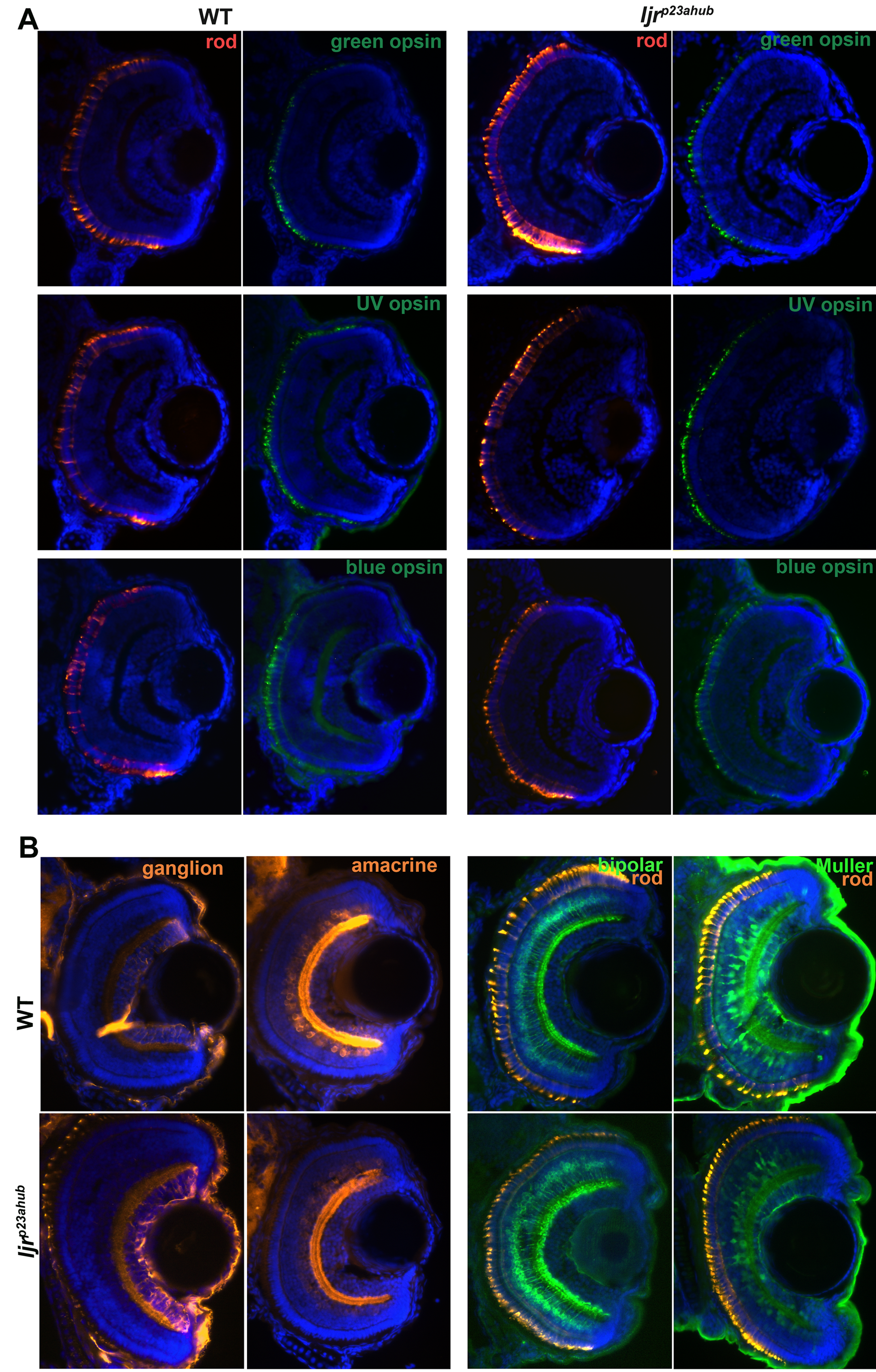

Supplement: S1 Fig — (A) No changes in green-, UV- and blue-sensitive cone opsin expression in ljrp23ahub mutants. Retinal cryosections from WT and ljrp23ahub embryos at 4 dpf immunolabeled for rods (4C12, red) and the green-, UV- and blue-sensitive cone opsins (green). Nuclei were counterstained with DAPI (blue); dorsal is up. No differences in the number or expression levels are detected for any of the opsin subtypes, except for the increased number of rods in ljrp23ahub mutants. (B) retinal cryosections from WT and ljrp23ahub embryos at 4 dpf immunolabeled for ganglion cells (Zn8), amacrine cell (5E11), bipolar cells (PKCα) and rods (4C12). No differences were observed for any labeled retinal cell types. (TIF) [file pgen.1005968.s001.tif]

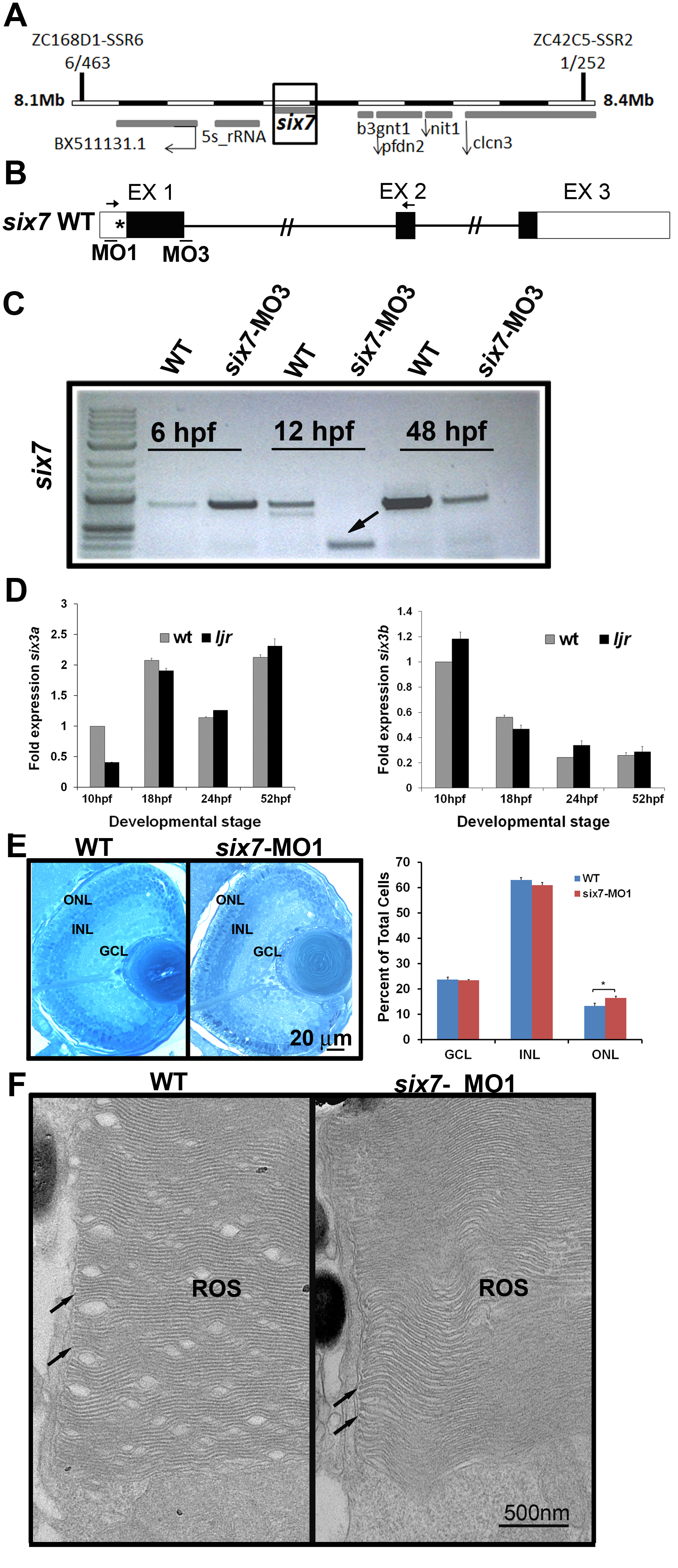

Supplement: S2 Fig — (A) Linkage analysis places the ljrp23ahub locus on chromosome 7, 6 out of 463 larvae show recombination at marker ZC168D1-SSR6 and 1 out of 252 larvae show recombination at marker ZC42C5-SSR2. (B) Diagram of predicted morpholino recognition sites (bars) in six7 loci. MO1 targets a translational site and MO3 blocks a donor splice site in intron 1 of six7. Incorrect splicing can be seen in morpholino-injected animals using primers in exon1 and 2 (arrows). (C) RT-PCR fragments, using primers highlighted in A, were analyzed by 1% agarose gel electrophoresis. Arrow highlights the six7 alternative spliced product obtained at 12 hpf (* indicates the new cryptic splice site). (D) No changes in the expression of the homologues six3a (left graph) or six3b (right graph) were detected between WT and ljrp23ahub mutants (n = 30 embryos per group). All the real-time PCR experiments were carried out in triplicates and normalized to β-actin. (E) Plastic sections of 4 dpf retinas from WT and six7-MO1 injected larvae. The three layers are regularly arranged in WT and morphant retinas. Close examination of the retina revealed a densely packed ONL in morphants. Graph showing the average number of nuclei per unit area (WT, n = 3, 2 sections each; six7-MO1, n = 3, 2 sections each). Cells are increased in the ONL of six7 morphants. Student t test, arcsine transformation, *p<0.05. (F) Electron micrographs of rod outer segments (ROS) from central retina of WT and six7-MO1 larvae. ROS from six7 morphants show their typical morphology of parallel- flattened sacs with continuously membranes at the edges (arrows) indistinguishable from WT ROS. (TIF) [file pgen.1005968.s002.tif]

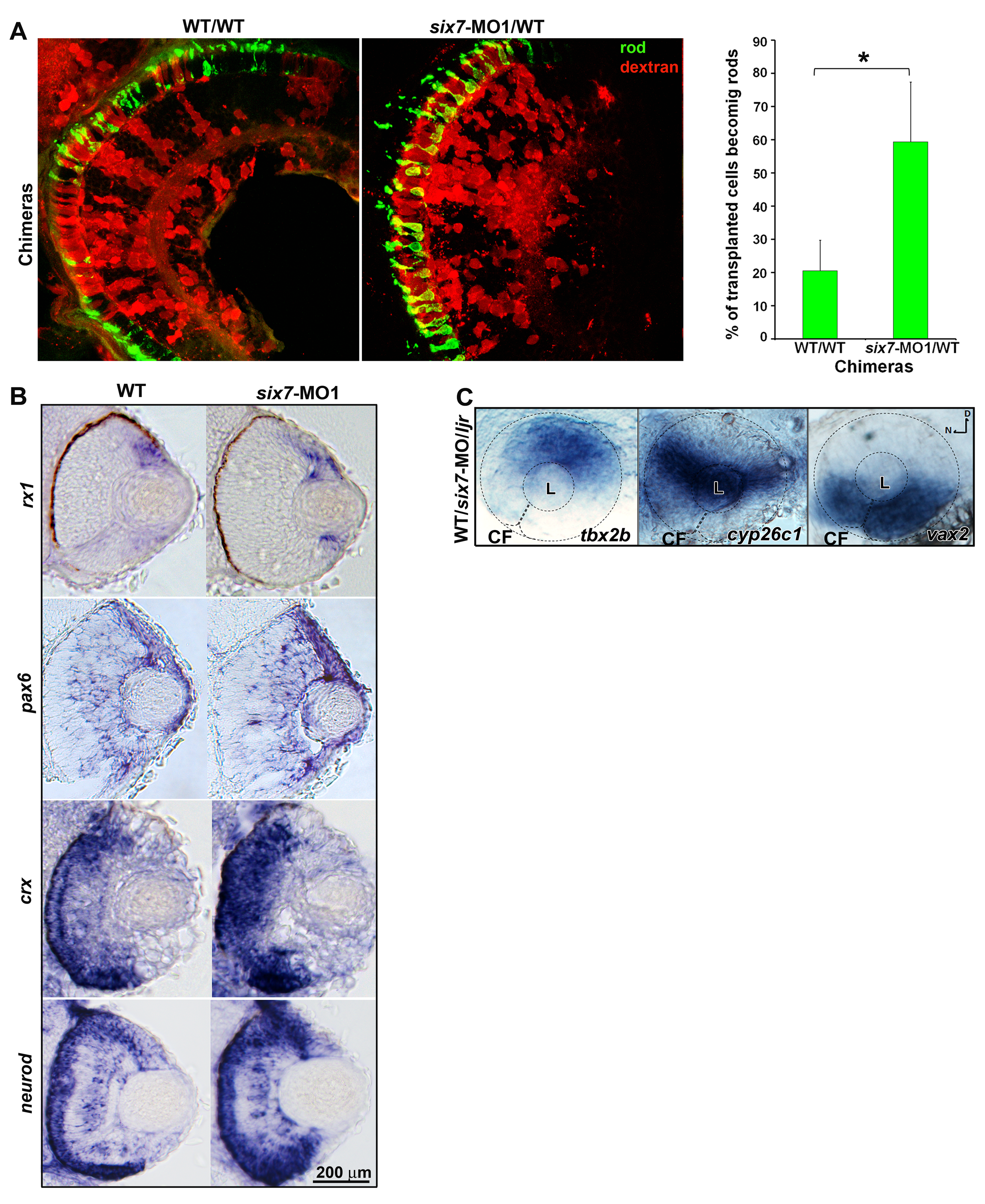

Supplement: S3 Fig — (A) Histological sections of chimera retinas labeled for rods (4C12, green). six7-MO donor cells (red) preferentially generate rods compare to WT donor cells. Note the gap in rod labeling in WT/WT controls. Graph represents the percentage of donor cells that differentiate as rods in the central retina of WT/WT (n = 5) and six7-MO1/WTchimeras (n = 6). *p<0.05, student t test. (B) rx1-, pax6a-, crx- and neurod-in situ hybridization (blue) in a retinal cryosection from 48-hpf WT and six7-MO1 embryos. No labeling of retinal progenitors cell markers (rx1 and pax6a) were observed in the ONL of six7-morphant retinas. (C) Whole-mount in situ hybridization for the dorsal (tbx2b), midline (cyp26c1) and ventral (vax2) retinal marker. Labeling was indistinguishable different between WT and mutant embryos. (TIF) [file pgen.1005968.s003.tif]

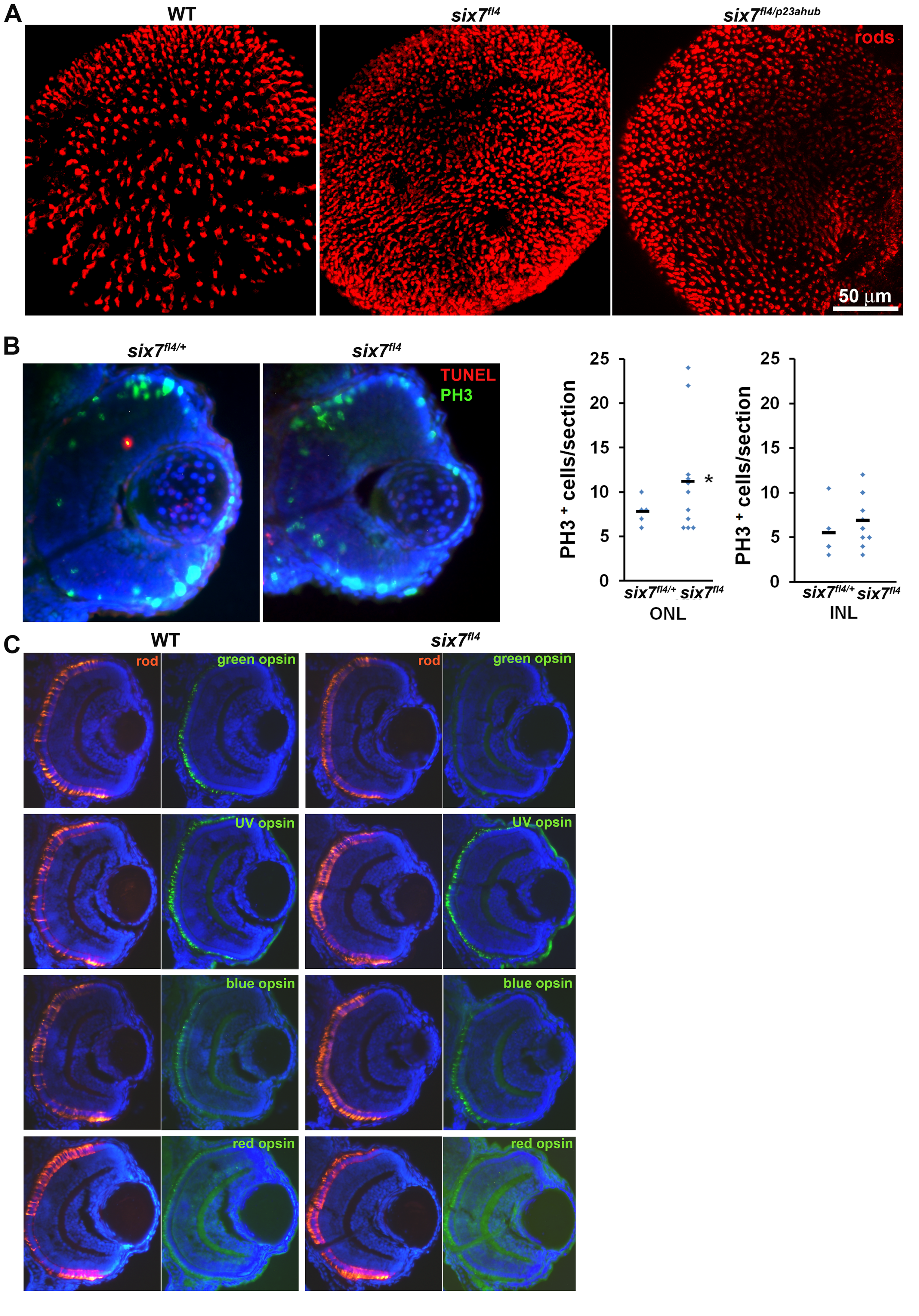

Supplement: S4 Fig — (A) Confocal immunofluorescent images labeled for rods (4C12, red) from WT, six7fl4 and six7fl4/p23ahub retinas at 4 dpf. (B) Retinal cryosections from carrier animals (six7fl4/+, n = 5, 1–2 sections/retina) and six7 (n = 11) embryos at 56 hpf co-labeled for TUNEL (red) and PH3 (green), nuclei counterstained with DAPI. No differences in TUNEL labeling were detected. Graphs showing the number of PH3+ cells by section (excluding CMZ). Number of PH3+ cells is significantly greater in ONL of six7fl4 mutants at 56 hpf. Un-paired Student t test with Welch’s correction, *p<0.05). (C) Retinal cryosections from WT and six7fl4 embryos at 4 dpf immunolabeled for rods (4C12, red) and the green-, UV-, blue- and red-sensitive opsins (green). Nuclei were counterstained with DAPI (blue). Dorsal is up. Depleted green-sensitive opsin expression is noticeable in six7fl4, but other opsins appear unaltered. (TIF) [file pgen.1005968.s004.tif]
