# Supplementary material for: Genetic Dissection of Dual Roles for the Transcription Factor six7 in Photoreceptor Development and Patterning in Zebrafish
Source: PLoS Genet. 2016 Apr 8;12(4):e1005968. doi: 10.1371/journal.pgen.1005968 (PMC4825938; doi:10.1371/journal.pgen.1005968)
Supplement: S1 Table — (RTF) [file pgen.1005968.s005.rtf]

Table S1. Primer sequences
Gene	Forward primer (5'          3')	Reverse primer (5'           3')	Use	
â-actin	TGGTATTGTGATGGACTCTGG	GCTGTAGCCACGCTCGGTC	RT-qPCR	
six7	GACCAGACTGGACCTCACG	GCAAATCAGACTGTTGTTGG	RT-qPCR	
six3a	CAAGCAATAGGGCAGAATGG	 TTGACGTGCCCGTGTCGAC	RT-qPCR	
six3b	TACAGACAGTGACTCTGATTTC	GTCAGTCCGTTGAAGCGCTG	RT-qPCR	
six7 exon 1- 2	GACCTACAGAGAATTCGTCC'	GAGCACAACCCACTCCTGC	PCR for MO3	
six7 spacer region 	TGCCGTCTGGAAGTTCTCC	ACTTCTCTACCGGGCCGAG	PCR TALENs	
six7	TGCCGTCTGGAAGTTCTCC	GCAAATCAGACTGTTGTTGG	probe	
vax2	TGACAGGAACGAACTTCGCTAG	TTCGGAGGTGGATGATGAGC	probe	
cyp26c1	GTTCGGGCACGATTTCTGCC	CTCTGATTCCCTTGCGCAGG	probe	
 ljr region	GATGTGAGATGTAAATCGGTC	CATATCGTTATCGCAGTACTC	PCR deletion	
six7	GACCTACAGAGAATTCGTCC	ACTTCTCTACCGGGCCGAG	PCR	
